# m^6^A methylation: a process reshaping the tumour immune microenvironment and regulating immune evasion

**DOI:** 10.1186/s12943-022-01704-8

**Published:** 2023-03-01

**Authors:** Xiaoxue Cao, Qishun Geng, Danping Fan, Qiong Wang, Xing Wang, Mengxiao Zhang, Lu Zhao, Yi Jiao, Tingting Deng, Honglin Liu, Jing Zhou, Liqun Jia, Cheng Xiao

**Affiliations:** 1 Institute of Clinical Medicine, China-Japan Friendship Hospital, Beijing, China; 2 Graduate School of Peking Union Medical College, Chinese Academy of Medical Sciences/Peking Union Medical College, Beijing, China; 3 Beijing Key Laboratory of Research of Chinese Medicine on Prevention and Treatment for Major Diseases, Experimental Research Center, China Academy of Chinese Medical Sciences, Beijing, China; 4 China-Japan Friendship Clinical Medical College, Beijing University of Chinese Medicine, Beijing, China; 5 China-Japan Friendship Hospital, Capital Medical University, Beijing, China; 6 Department of Physiology, School of Basic Medical Sciences, Guangxi Medical University, Nanning, Guangxi China; 7 Oncology Department of Integrated Traditional Chinese and Western Medicine, China-Japan Friendship Hospital, Beijing, China; 8 Department of Emergency, China-Japan Friendship Hospital, Beijing, China

**Keywords:** N6-methyladenosine (m^6^A), Tumour immune microenvironment, Hypoxia, Metabolic reprogramming, Acidity, Immune suppression, Immune cells, Immune evasion, Immune therapy

## Abstract

N6-methyladenosine (m^6^A) methylation is the most universal internal modification in eukaryotic mRNA. With elaborate functions executed by m^6^A writers, erasers, and readers, m^6^A modulation is involved in myriad physiological and pathological processes. Extensive studies have demonstrated m^6^A modulation in diverse tumours, with effects on tumorigenesis, metastasis, and resistance. Recent evidence has revealed an emerging role of m^6^A modulation in tumour immunoregulation, and divergent m^6^A methylation patterns have been revealed in the tumour microenvironment. To depict the regulatory role of m^6^A methylation in the tumour immune microenvironment (TIME) and its effect on immune evasion, this review focuses on the TIME, which is characterized by hypoxia, metabolic reprogramming, acidity, and immunosuppression, and outlines the m^6^A-regulated TIME and immune evasion under divergent stimuli. Furthermore, m^6^A modulation patterns in anti-tumour immune cells are summarized.

## Background

The tumour immune microenvironment (TIME) comprises intratumour immunological components and orchestrates tumour immunity [1]. The high heterogeneity within the TIME makes immunotherapy challenging [2, 3]. Tumours exploit and reshape the TIME to avoid immune surveillance [4, 5]. Hence, harnessing the TIME to boost anti-tumour immunity has been a core strategy of immunotherapy [6, 7]. Epigenetic modification has been extensively investigated in tumours and has a pivotal role in tumour immunoediting [8–10]. The regulatory role of DNA methylation in tumour immunity has been well characterized over the past few years [11]. Recent evidence has moved forwards to the role of diverse RNA methylation mechanisms in TIME modulation and revealed their utility as promising targets in immunotherapy [12, 13].

**Fig. 1:**
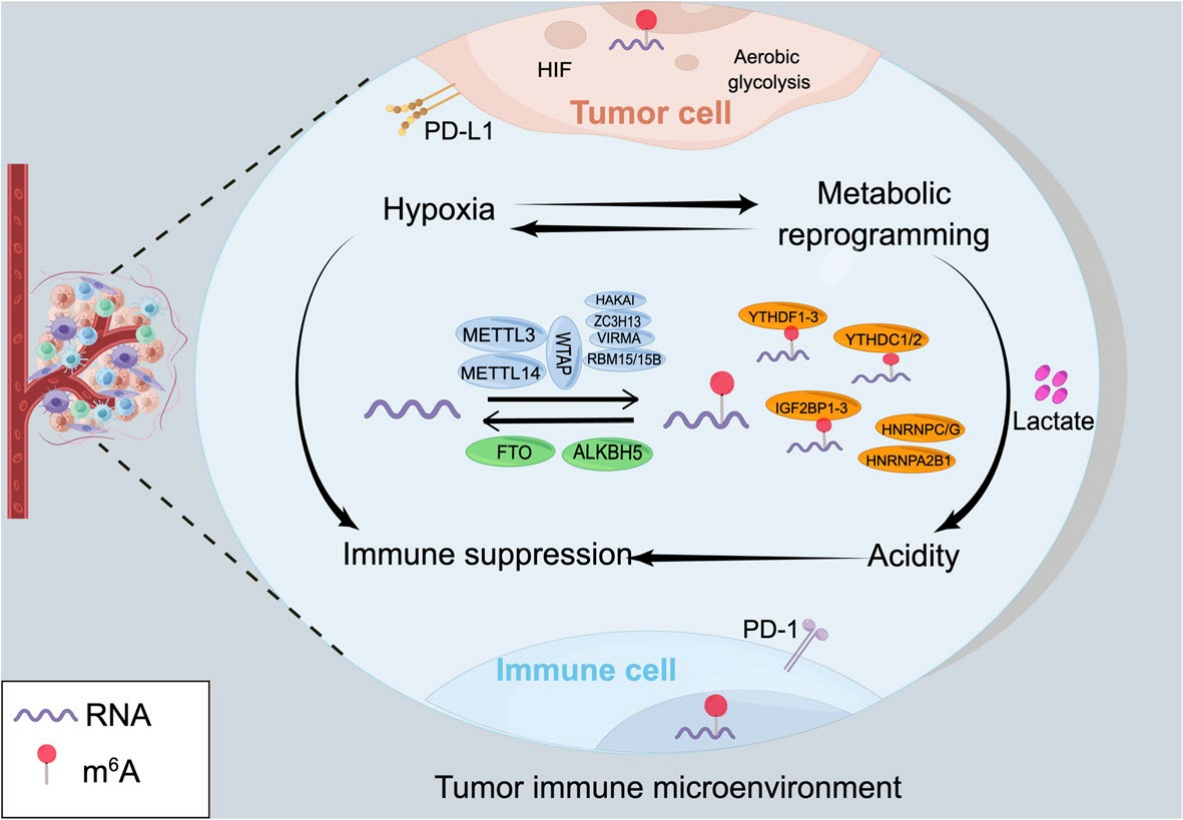
Schematic diagram of the topic of this review. The tumour immune microenvironment (TIME) is characterized by hypoxia, metabolic reprogramming, acidity, and immune suppression. There is potential crosstalk between hypoxia and metabolic reprogramming (HIF signalling can induce tumour metabolic reprogramming, and altered metabolism affects oxygen consumption in tumours). Accumulated lactate generated by high aerobic glycolysis forms an acidic TIME. Hypoxia, metabolic reprogramming, and acidity promote immune suppression and lead to tumour immune evasion. This review provides an overview of m^6^A methylation patterns in the TIME and immune cells and the role of m^6^A methylation in tumour immune escape

Although more than 170 types of chemical RNA modifications have been identified since the first discovery in the 1970s [14], the biological functions of RNA methylation were gradually revealed until the last decade [15, 16]. N6-methyladenosine (m^6^A) RNA methylation is the most prevalent internal mRNA modification in eukaryotic cells. With the development of transcriptome-wide sequencing techniques, the role of m^6^A methylation in many cellular functions and disease pathologies has been well-characterized [17–19]. Recently, aberrant m^6^A RNA modification has been identified in various tumours [20], participating in tumorigenesis, metastasis, chemo- and radioresistance [21–23]. Growing evidence has revealed an emerging role of m^6^A methylation in regulating the TIME and tumour immunity [24, 25]. Previous reviews have provided a clear and systematic summary of m^6^A regulation in various tumours and immune cells, as well as the application of strategies related to m^6^A methylation in cancer prognostication and therapy [26–29]. This review outlines the regulatory role of m^6^A modification in the intricate TIMEs, including hypoxia, metabolic reprogramming, acidity, and immune suppression, and further describes the latest research progress related to m^6^A modification-facilitated tumour immune evasion in diverse TIMEs. Furthermore, m^6^A methylation patterns in several tumour-infiltrating immune cells are summarized (Fig. 1). Finally, a few future prospects on m^6^A methylation are also listed in the last section. Thus, this review provides an overview of m^6^A-regulated tumour immune surveillance and novel insights into immunotherapy.

## m^6^A RNA modification

m^6^A RNA modification is a critical posttranscriptional mechanism that regulates RNA metabolism and biological functions [30]. With a dynamic and reversible process modulated by 3 types of proteins, writers, erasers, and readers, the functional effects of m^6^A methylation are accomplished. m^6^A writers install methyl groups on target RNA, while erasers remove the methyl group from RNA to maintain the reversible process. Through m^6^A modification, marked RNAs are specifically recognized and tethered by m^6^A readers. These RNA-binding proteins perform distinct functions, affecting RNA splicing, nuclear export, stability, translation, and degradation, thus regulating gene expression [31]. Aberrant m^6^A methylation in tumour development promotes oncogenes expression in a context-dependent manner, driving the tumour-promoting effect [32, 33]. In addition to performing the functional effects in tumour development, m^6^A methylation can also be altered by tumour therapy. The m^6^A deposition occurs at UV-induced DNA damage sites within 2 min in response to UV irradiation, facilitating the recruitment of DNA polymerase κ for DNA repair and cell survival, which indicates a crucial role of dynamic m^6^A RNA modification in tumour radioresistance as well as a potential target for improving radiotherapy [34].

### m^6^A writers

m^6^A writers are methyltransferases responsible for the installation of methyl groups on target RNA. Canonical methyl addition is catalysed by a writer complex comprised of multifunctional subunits. Methyltransferase-like 3 (METTL3) and methyltransferase-like 14 (METTL14) form a heterodimer as the writer complex core; the former possesses catalytic ability, while METTL14 allosterically activates METTL3 and facilitates RNA binding [35]. Additional adaptors and interactors are assembled into the writer complex. William tumour 1-associated protein (WTAP) is essential for nuclear localization of the complex core and m^6^A formation in mammalian cells. Two paralogous RNA binding motif proteins, RNA binding motif protein 15 (RBM15) and RBM15B, are reported to interact with METTL3 in a WTAP-dependent manner, contributing to methylation specificity. Other components, including zinc-finger CCCH domain-containing protein 13 (ZC3H13), vir-like m^6^A methyltransferase associated (VIRMA, also known as KIAA1429), and HAKAI, have been identified as WTAP interactors that are essential to m^6^A formation [36]. m^6^A methylation has sequence specificity, and METTL3/14 complex-mediated m^6^A methylation is preferentially enriched at RRACH (R = A or G; H = A, C, or U) motifs [37], which are widely used to identify m^6^A methylation.

### m^6^A erasers

m^6^A erasers mediate the removal of internal m^6^A from RNA, facilitating reversible, dynamic m^6^A methylation to modulate complex processes. M^6^A demethylation is catalysed by 2 enzymes, fat-mass and obesity-associated protein (FTO) and alpha-ketoglutarate-dependent dioxygenase ALKB homologue 5 (ALKBH5). FTO was the first identified demethylase and mediates the demethylation of m^6^A in mRNA and U6 RNA [38]. ALKBH5 has been discovered to function as an exclusive m^6^A regulator rather than modulating other types of RNA modifications; it demethylates specific transcripts at the 3’UTR and expedites mRNA export from the nucleus to the cytoplasm. Growing evidence has shown that FTO- and ALKBH5-mediated m^6^A demethylation is involved in various tumours [39–42], displaying biological context-dependent functions. FTO is largely enhanced in some leukaemia cells and promotes leukaemia progression through multiple signalling pathways [43, 44]. FTO also serves as an oncogene in other tumours, such as melanoma [45] and breast cancer [46]. ALKBH5 expression is induced in hypoxia and facilitates HIF-regulated biological events and diseases [47, 48].

### m^6^A readers

m^6^A readers are RNA-binding proteins with distinct regulatory effects. By specifically recognizing and anchoring m^6^A-bearing RNAs, reader proteins affect RNA splicing, nuclear transport, stability, translation, and RNA decay [49]. The YTH domain-containing proteins, including YTHDF1-3 and YTHDC1-2, were the first identified RNA-binding proteins (RBPs), the YTH domain is responsible for specific RNA binding. The cytosolic YTHDF family possesses distinct functions. YTHDF1 promotes RNA translation through interaction with eIFs [50]. YTHDF2 is the dominant protein responsible for selective RNA decay [51]. YTHDF3 cooperates with YTHDF1 and YTHDF2 to accelerate either translation or degradation of target transcripts. YTHDF3 deletion decreased the RNA binding specificity of YTHDF1-2 [52]. This dynamic and integrated function of the DF family represents a delicate regulatory mechanism of m^6^A methylation. The nuclear reader YTHDC1 binds to SRSF3 to regulate RNA splicing and nuclear export [53]. YTHDC2 regulates RNA translation and stability, exerting various regulatory effects [54–56]. YTHDC2 is upregulated in several cancers but possesses a tumour-suppressive role in lung cancer [57, 58]. Insulin-like growth factor 2 mRNA-binding proteins (IGF2BP1-3) are a class of m^6^A binding proteins that promote the stability and expression of target genes, exhibiting oncogenic activity in tumours [59]. Additionally, cytosolic METTL3 also serves as a reader, enhancing mRNA translation through interaction with the eukaryotic translation initiation factor eIF3h [60].

## m^6^A methylation regulates TIME reprogramming and affects immune evasion

Cancer cells reshape the TIME through multiple mechanisms, leading to rapid proliferation and escape from host immune surveillance [61, 62]. The altered TIME is characterized by increased oxygen consumption, nutrient deprivation, accumulation of metabolites, and immune dysregulation, which form an extracellular niche with hypoxia, metabolic reprogramming, and immune suppression, facilitating tumour immune evasion and leading to poor efficacy of immunotherapy [63]. Recent evidence has highlighted that m^6^A methylation is a mechanism that is indispensable in reshaping the TIME and regulating tumour immune surveillance.

### m^6^A methylation responds to a hypoxic TIME and favours immune evasion

Hypoxia is one of the detrimental hallmarks of solid tumours and is generated from the augmentation of oxygen consumption and abnormal vascularization in the tumour context [62, 64]. Hypoxia-inducible factors (HIFs) are transcriptional activators and principal regulators that regulate target genes at the transcriptional and translational levels to induce tumorigenesis, angiogenesis, metastasis, and metabolic adaptation under hypoxic stress [65–67]. An increasing number of investigations have unravelled the mechanisms of HIF-driven tumour immunity and immune evasion [68–70]. HIFs hinder the type I IFN pathway and facilitate immune suppression in numerous tumours [71]. HIF-1α enhances programmed death-ligand 1 (PD-L1) expression on myeloid-derived suppressor cells (MDSCs), macrophages, and tumour cells, impairing T-cell activation [72]. In hepatocellular carcinoma (HCC), HIF-1 enhances the enrichment of MDSCs [73]; additionally, HIF-2α deletion in cancer-associated fibroblasts in pancreatic cancer reduces tumour infiltration of immune-suppressive immune cells, including M2 TAMs and Tregs, restoring the efficacy of immunotherapy [74]. Recent evidence has revealed that hypoxia in brain tumours restrains the anti-tumour immunity of γδ T cells [75]. Notably, hypoxia is a variable factor that directs epigenetic alterations in tumours, and the modulation of hypoxia in the TIME is closely linked to epigenetic reprogramming [76, 77].

**Fig. 2:**
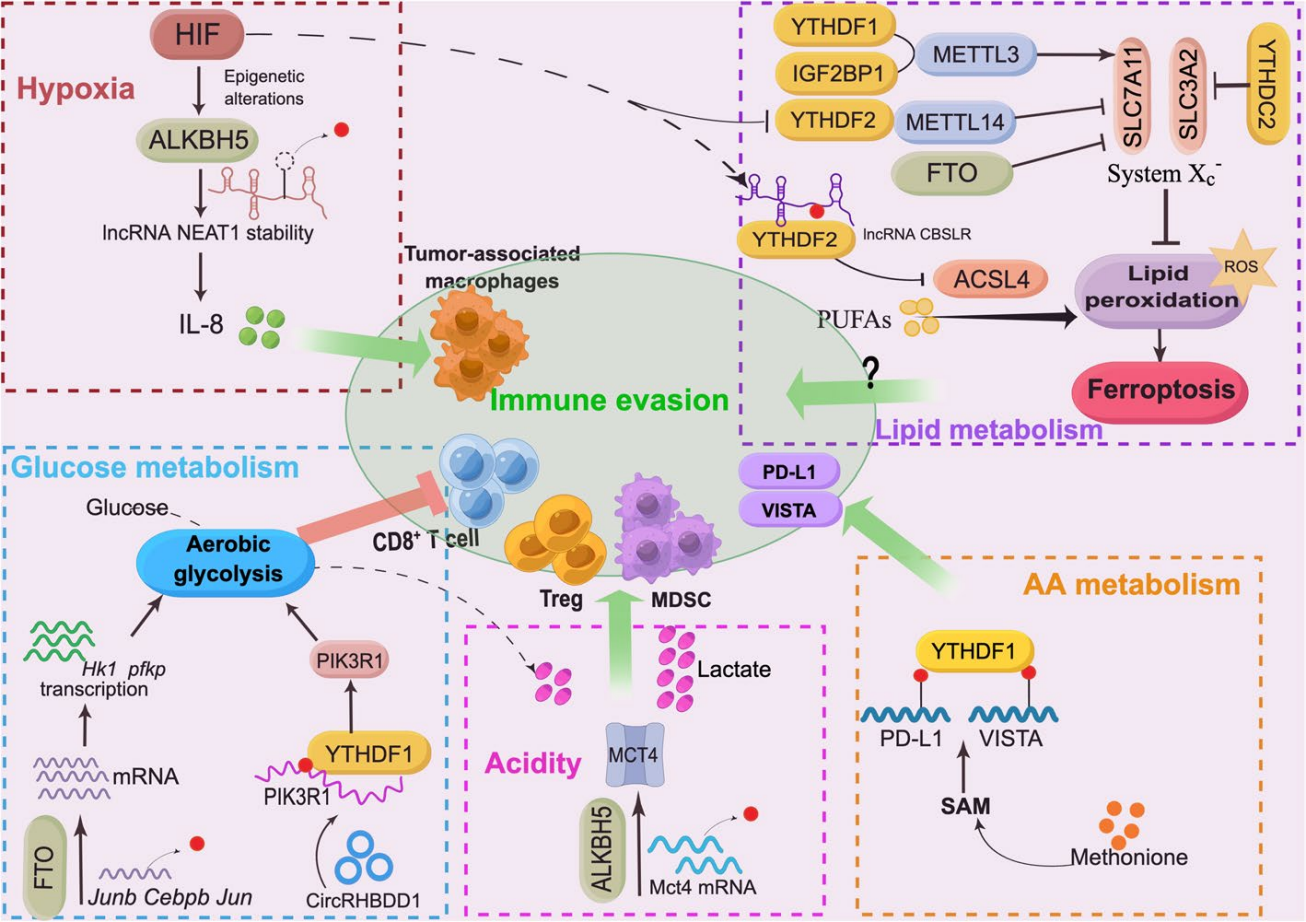
m^6^A methylation reshapes the TIME and affects tumour immune evasion under divergent factors. The predominant driving factors in the TIME include hypoxia, metabolic reprogramming, and acidity. PUFAs, polyunsaturated fatty acids

Recent evidence has shown that key m^6^A enzymes are distinctly regulated in various hypoxic cancer cells and revealed the critical role of m^6^A modification in hypoxia-mediated tumour progression, malignancy, and chemoresistance. Downregulated METTL3 expression in the hypoxic TIME weakens the sensitivity of HCC cells to sorafenib by reducing YTHDF1-promoted transcriptional stability of FOXO3 [78] (Table 1). ALKBH5 and FTO exhibit distinct regulatory roles in hypoxic tumours. Increased ALKBH5 expression induced by hypoxia maintains the stem-like cell phenotype and tumorigenicity of breast cancer stem cells (BCSCs) and endometrial cancer stem-like cells (ECSCs) in the hypoxic TIME in an m^6^A-dependent manner [79, 80]. Decreased FTO expression induced by hypoxia facilitated colorectal cancer (CRC) metastasis by inhibiting IGF2BP3-facilitated upregulation of metastasis-associated protein 1 (MTA1) [81]. These results reveal an oncogenic role of ALKBH5 and a tumour-suppressive role of FTO in hypoxic tumours. m^6^A reader proteins are context-dependent effectors of m^6^A methylation. Indeed, numerous studies have indicated multiple regulatory effects of m^6^A readers in hypoxia-driven tumours. Hypoxia can reprogram the TIME through autophagy, and a recent study found that the induction of YTHDF1 expression by HIF-1α contributed to the induction of autophagy and autophagy-related HCC progression [82]. YTHDF2 upregulation by HIF-1α promotes cancer cell proliferation in acute myeloid leukaemia (AML) [83] and lung squamous carcinogenesis (LUSC) [84]. Another study showed that HIF-2α-restrained YTHDF2 stimulated inflammation and impaired vascular normalization in HCC through the disruption of YTHDF2-facilitated decay of IL-11 and serpin family E member 2 (SERPINE2) mRNA [85]. Hypoxia-induced lncRNA KB-1980E6.3 recruited IGF2BP1, which further increased the stability of c-Myc mRNA, facilitating self-renewal of BCSCs and tumorigenesis [86]. IGF2BP3 knockdown in stomach cancer cells inhibited hypoxia-driven cell migration and angiogenesis by downregulating HIF-1α expression [87]. m^6^A methylation-modulated hypoxic tumours also dictate metabolic reprogramming, as described in the next section. These data provide solid evidence for the possibility of reversible and dynamic modulation of the m^6^A-regulated hypoxic tumour environment and novel insights into tumour therapy. Although there are no direct data on m^6^A-mediated immunoregulation, these investigations have led us to further explore the regulatory role of m^6^A in the hypoxic TIME and immunotherapy. Notably, the latest research links m^6^A methylation to a hypoxia-driven TIME phenotype in glioblastoma (GBM) and suggests that m^6^A methylation is involved in immune evasion. Dong and his colleagues found that ALKBH5 expression induced by hypoxia promoted the recruitment of TAMs in GBM and an immunosuppressive microenvironment in allograft tumours [88]. ALKBH5-mediated removal of m^6^A deposition from the lncRNA NEAT1 enhanced transcript stability and NEAT1-mediated paraspeckle assembly, which resulted in the relocation of the transcriptional repressor SFPQ from the CXCL8 promoter to paraspeckles, increasing CXCL8/IL8 expression and contributing to TAM enrichment and tumour progression. That study illustrated the mechanism by which ALKBH5 facilitates an immunosuppressive TIME in the hypoxic GBM niche, providing novel insights into m^6^A-guided immunotherapy (Fig. 2). In line with previous studies, these data suggest a tumour-promoting role of ALKBH5. Based on the critical role of hypoxia in immune escape, much clarification of the epigenetic reprogramming that occurs in the hypoxic TIME and its role in tumour immunity is needed.

**Table 1:** m^6^A modulation under diverse factors in the TIME

Factor in the TIME	m^6^A regulator	Cancer type	Molecular mechanism	Effect on tumours and immune surveillance	Reference
Hypoxia	METTL3	HCC	HIF-METTL3-FOXO3 axis, enhancing FOXO3 mRNA stabilization by YTHDF1	Promoting sorafenib resistance	[78]
	ALKBH5	BCSC	HIF-ALKBH5-NANOG axis, enhancing NANOG mRNA and protein level	Promoting cancer stem-cell like phenotype and tumorigenesis	[79]
	ALKBH5	ECSC	HIF-ALKBH5-SOX2 axis, enhancing SOX2 transcription	Promoting cancer stem-cell like phenotype and tumorigenesis	[80]
	FTO	CRC	Repressing MTA1 mRNA stability by IGF2BP2	Inhibiting cancer metastasis and progression	[81]
	YTHDF1	HCC	Enhancing ATG2A and ATG14 translation	Enhancing autophagy and autophagy-steered HCC progression	[82]
	YTHDF2	AML	AML1/ETO-HIF1α-YTHDF2 axis, decreasing total m^6^A level	Promoting cancer proliferation	[83]
	YTHDF2	LUSC	Activating of mTOR/AKT	Promoting tumorigenesis and invasion, inducing EMT	[84]
	YTHDF2	HCC	HIF-2α-YTHDF2, preventing IL-11 and SERPINE2 RNA decay	Inducing inflammation-driven malignancy and disrupting of vascular normalization	[85]
	IGF2BP1	BCSC	Hypoxic lncRNA KB-1980E6.3/IGF2BP1/c-Myc axis, inducing c-Myc mRNA stability	Promotion of self-renewal and tumorigenesis	[86]
	IGF2BP3	SC	Inducing of HIF1A mRNA expression	Retaining HIF-mediated cell migration and angiogenesis	[87]
	ALKBH5	GBM	Enhancing lncRNA NEAT1 transcript stability	Promoting IL8 expression, leading to TAM enrichment and immunosuppressive TIME	[88]
Glycolysis reprogramming	METTL3	CC	Enhancing HK2 mRNA stability through YTHDF1	Promoting glycolysis and tumorigenesis	[89]
	METTL3	CRC	METTL3/LDHA axis, enhancing LDHA transcription and translation through HIF-1 alfa and YTHDF1, respectively.	Inducing 5-FU chemoresistance	[90]
	METTL3	CRC	Stabilizing transcripts of HK2 and GLUT1 through IGF2BP2, IGF2BP3, respectively	Promoting tumour glycolysis and cancer progression	[91]
	METTL3	CC, HCC	m^6^A/PDK4 axis, enhancing PDK4 translation and mRNA stability through YTHDF1/eEF-2 and IGF2BP3	Promoting tumour glycolysis and cancer progression	[92]
	METTL3, ALKBH5	LUAC	Enhancing ENO1 translation through YTHDF1	Promoting tumour glycolysis and tumorigenesis	[93]
	METTL14	HCC	METTL14-USP48-SIRT6 axis, enhancing USP48 mRNA stability	Attenuating tumour glycolysis and malignancy	[94]
	METTL14	RCC	Repressing BPTF mRNA stability	Inhibiting tumour glycolysis and distal lung metastasis	[95]
	METTL14	GC	Repressing LHPP expression	Promoting tumour glycolysis and cancer progression	[96]
	WTAP	BCSC	ERK1/2-WTAP-ENO1, increasing ENO1 mRNA stability	Promoting tumour glycolysis and cancer progression	[97]
	WTAP	GC	Enhancing stability of HK2 mRNA	Promoting tumour glycolysis and cancer progression	[98]
	KIAA1429	CRC	Enhancing stability of HK2 mRNA	Promoting tumour glycolysis and cancer progression	[99]
	FTO	AML	FTO/PFKP/LDHB axis, upregulating the expression of PFKP and LDHB.	Promoting tumour aerobic glycolysis	[100]
	FTO	PTC	Decreasing stability of APOE mRNA by IGF2BP2	Attenuating tumour glycolysis and cancer growth	[101]
	FTO	LUAC	Wnt/β-catenin/FTO/c-Myc, FTO inhibits MYC mRNA translation	Inhibiting tumour glycolysis and tumorigenesis	[102]
	ALKBH5	Bca	Suppressing CK2α mRNA stability	Suppressing tumour glycolysis and cisplatin resistance	[103]
	IGF2BP2	CRC	LINRIS-IGF2BP2-MYC axis, enhancing MYC mRNA stability	Promoting tumour glycolysis and cancer progression	[104]
	YTHDF1	BC	YTHDF1-PKM2, upregulating PKM2 expression	Promoting tumour glycolysis, cancer growth and metastasis	[105]
	YTHDF3	PC	Promoting LncRNA DICER-AS1 degradation	Promoting tumour glycolysis	[106]
	YTHDC1	PDAC	YTHDC1/miR-30d/RUNX1axis, promoting miR-30d and repressing RUNX1.	Attenuating tumour glycolysis and tumorigenesis	[107]
	FTO	Melanoma, NSCLC	Enhancing transcripts of c-Jun, JunB, and C/EBPβ	Promoting tumour glycolysis and immune evasion	[108]
	YTHDF1	HCC	CircRHBDD1/YTHDF1/PIK3R1 axis, enhancing PIK3R1 translation	Promoting tumour glycolysis and restraining PD-1 therapy	[109]
Lipid metabolism reprogramming	m^6^A writer complex, m^6^A erasers	HCC	m^6^A methylation negatively regulates CES2 expression by YTHDC2.	m^6^A methylation augment increased lipid accumulation	[110]
	METTL3	HCC	Enhancing lncRNA LINC00958 stability	Promoting lipogenesis and tumour progression	[111]
	FTO	EC	Enhancing HSD17B11 expression by YTHDF1	Promoting lipid droplets and tumour development	[112]
	METTL3, METTL14	CRC	Impairing of RNA decay of DEGS2, promoting DEGS2 levels	Inducing lipid dysregulation, tumour progression	[113]
	METTL3	GBM	Stimulating SLC7A11 mRNA splicing and maturation	Inhibiting ferroptosis	[114]
	METTL3	HB	m^6^A/IGF2BP1/SLC7A11 axis, preventing SLC7A11deadenylation.	Enhancing ferroptosis resistance	[115]
	METTL3	LUAC	Stabilizing SLC7A11 m^6^A modification	Promoting tumour growth and inhibiting ferroptosis	[116]
	FTO	PTC	Promoting SLC7A11 downregulation	Promoting ferroptosis	[117]
	METTL14	HCC	HIF-1α/METTL14/YTHDF2/SLC7A11 axis, disrupting METTL14 mediated SLC7A11 silencing by YTHDF2	Inhibiting ferroptosis	[118]
	YTHDC2	LUAC	YTHDC2/HOXA13/SLC3A2 axis, destabilizing HOXA13 mRNA and inhibiting SLC3A2 expression	Inducing ferroptosis	[119]
	YTHDF2	GC	CBSLR/YTHDF2/CBS signalling, decreasing the stability of CBS mRNA and promoting ACSL4 degradation	Inhibiting ferroptosis	[120]
AA metabolism reprogramming	FTO	CRC	FTO/YTHDF2/ATF4, disrupting ATF4 RNA decay by YTHDF2	Promoting autophagy activation and compromising antitumour effect	[121]
	FTO	ccRCC	Enhancing SLC1A5 expression	Promoting tumour growth and survival	[122]
	YTHDF1	CRC	Enhancing GLS1 synthesis.	Promoting glutamine uptake and cisplatin resistance	[123]
	YTHDF1	CRC	Dietary methionine and YTHDF1 promotes m^6^A methylation and translation of PD-L1 and VISTA	Inhibiting antitumour immunity.	[124]
Acidity	ALKBH5	Melanoma, CRC	Enhancing Mct4 mRNA levels	Enhancing extracellular lactate content and the promoting Treg and MDSC enrichment	[125]
	METTL3	CRC	METTL3/m^6^A/JAK/STAT3 axis, METTL3 lactylation promotes JAK translation.	Promoting immunosuppression of tumour-infiltrating myeloid cells.	[126]
Immune suppression	METTL3	BCa	JNK/METTL3/PD-L1, enhancing PD-L1 mRNA stability by IGF2BP1	Promoting tumour immune escape	[127]
	METTL3	BC	METTL3/IGF2BP3 axis, promoting stabilization of PD-L1 mRNA	Inhibiting immune surveillance	[128]
	METTL3	NSCLC	YTHDC1/circIGF2BP3, promoting circularization of circIGF2BP3	Facilitating PD-L1 deubiquitination and promoting immune escape	[129]
	METTL14	CCA	METTL14/Siah2/PD-L1 axis, enhancing Siah2 degradation by YTHDF2	Inhibiting PD-L1 ubiquitination and immune surveillance	[130]
	METTL14	HCC	METTL14/MIR155HG/PD-L1 axis, stabilizing MIR155HG	Facilitating PD-L1 upregulation and tumour immune escape	[131]
	ALKBH5	ICC	ALKBH5/PD-L1, preventing YTHDF2-mediated PD-L1 RNA decay	Sustaining PD-L1 expression and inhibiting immune surveillance	[132]
	ALKBH5	HNSCC	ALKBH5/RIG-I/IFNα axis, inhibiting mRNA maturation of DDX58 that encodes RIG-1	Inhibiting RIG-I-mediated IFNα secretion and promoting tumour progression	[133]
	FTO	AML	FTO/m^6^A/LILRB4, enhancing LILRB4 expression	Promoting cancer stem cell self-renewal and immune escape	[134]
	FTO	Melanoma	Preventing YTHDF2-mediated mRNA decay of PD-1, CXCR4, SOX10	Promoting anti-PD-1 resistance	[135]
	FTO	OSCC	Promoting the stability and expression of PD-L1 transcripts	Promoting immune resistance and tumour progression	[136]
	METTL3	CRC	m^6^A-BHLHE41-CXCL1/CXCR2 Axis, increasing CXCR1 transcription through m^6^A-promoted BHLHE41 expression.	Enhancing MDSCs migration and inhibiting CD8^+^T cells	[137]
	METTL3, YTHDF2	GBM	Active YY1–CDK9 transcription elongation complex enhanced METTL3, YTHDF2 levels	Promoting immune suppressive TIME	[138]
	ALKBH5	HCC	ALKBH5/MAP3K8 axis, enhancing MAP3K8 expression	Promoting tumour growth, metastasis and PD-L1^+^macrophage recruitment	[139]

*m*^*6*^*A* N6-methyladenosine, *HCC* Hepatocellular carcinoma, *BCSC* Breast cancer stem cell, *ECSCs* Endometrial cancer stem cells, *CRC* Colorectal cancer, *AML* Acute mygeeloid leukaemia, *LUSC* Lung squamous carcinogenesis, *SC* Stomach cancer, *GBM* Glioblastoma, *CC* Cervical cancer, *CRC* Colorectal cancer, *LUAC* Lung adenocarcinoma, *RCC* Renal carcinoma, *GC* Gastric cancer, *PTC* Papillary thyroid carcinoma, *BCa* Bladder cancer, *BC* Breast cancer, *PC* Pancreatic cancer, *PDAC* Pancreatic ductal adenocarcinoma, *NSCLC* Non-small cell lung carcinoma, *EC* Esophageal cancer, *HB* Hepatoblastoma, *ccRCC* Renal clear cell carcinoma, *ICC* Intrahepatic cholangiocarcinoma, *HNSCC* Head and neck squamous cell carcinoma, *OSCC* Oral squamous cell carcinoma

### m^6^A methylation regulates metabolic reprogramming in the TIME and immune escape

Metabolic reprogramming is a primary mechanism for tumour immune evasion [63]. In the TIME, proliferating cancer cells compete for nutrients with immune cells, and this nutrient restriction hampers the function and anti-tumour immunity of immune cells, favouring immune evasion. Additionally, metabolic alterations in glucose, lipid, and amino acid (AA) levels induce accumulations of metabolites that serve as checkpoints to regulate immune responses in multiple metabolic pathways [140]. Extensive evidence has shown that m^6^A modification is widely involved in metabolic reprogramming and affects tumorigenesis, metastasis, and chemoresistance.

#### Glucose metabolism

Glucose metabolism is the most essential energy process for tumours. Tumour cells preferentially choose glycolysis pathways over oxidative phosphorylation (OXPHOS) for rapid growth (the Warburg effect) [141]. Metabolic alterations are also critical for immunoregulation in the TIME [142] and dictate the function of immune cells. For instance, significantly increased glycolytic metabolism with downregulation of the OXPHOS pathway is present in activated T cells, dendritic cells (DCs), natural killer (NK) cells, and M1 macrophages as well as neutrophils [143]. Hence, glycolytic metabolism is essential for maintaining adaptive and humoral immunity, and tumour-driven glucose restriction in the TIME dampens the function of tumour-infiltrating immune cells, leading to immune escape [144–146]. Moreover, a hypoxic TIME prompts tumour glycolytic activity and drives an immune-excluded TIME [147, 148]. Targeting glycolysis to reprogram metabolism in tumours and immune cells has been considered a promising strategy for combination therapy to improve the efficacy of immune checkpoint blockades (ICBs) [149].

Studies have shown that m^6^A methylation facilitates glycolytic reprogramming through modulation of the expression of multiple glycolysis-related genes and signalling pathways in various tumours. METTL3-mediated m^6^A modification has a positive regulatory effect on glycolysis in numerous tumours, although it acts on different pathways [89–93]. METTL14 has a distinct glycolysis-regulating effect in tumours. METTL14 impedes HCC cell glycolysis through the METTL14-USP48-SIRT6 axis [94]. METTL14 deficiency in renal cell carcinoma (RCC) promotes distal lung metastasis by enhancing glycolytic reprogramming [95]. However, a glycolysis-promoting role of METTL14 through the repression of LHPP, which inhibits cancer cell metabolism, has been reported in gastric cancer (GC) [96]. Other m^6^A methyltransferases, including WTAP and KIAA1429, have been found to have a glycolysis-promoting role in cancers [97–99]. The m^6^A demethylase FTO regulates multiple key enzymes and pathways that control aerobic glycolysis in distinct cancer cells [100–102]. ALKBH5 suppresses casein kinase 2 (CK2) α-mediated glycolysis in bladder cancer (BCa), enhancing the sensitivity of tumour cells to cisplatin [103]. The m^6^A reader protein IGF2BP2 (IMP2) is responsible for RNA stability and exhibits a role in facilitating tumour glycolytic reprogramming by stabilizing effectors that promote aerobic glycolysis [104]. m^6^A binding proteins of the YTH family modulate the Warburg effect depending on their functions. YTHDF1 plays a cancer-promoting role and enhances glycolysis by upregulating PKM2 and HK2 mRNA levels in an m^6^A-dependent manner [89, 105]. YTHDF3 has been reported to have a glycolysis-promoting effect in pancreatic cancer because it drives the degradation of the lncRNA DICER-AS1, which inhibits glycolytic metabolism [106]. Another study revealed that YTHDC1 stimulates the cancer suppressor miR-30d, attenuating glycolysis in pancreatic ductal adenocarcinoma [107] (Table 1). Mounting evidence indicates that glycolytic reprogramming in cancers is widely modulated by m^6^A modification. As such, how does m^6^A-driven metabolic reprogramming affect anti-tumour immunity and immunotherapy? Recent studies have provided evidence to answer this question. Xu and her colleagues discovered that tumour-derived FTO dampens CD8^+^T cell activation and effector function by rewiring tumour glycolysis. FTO inhibition by the small molecule Dac51 removes metabolic barriers in T cells and blocks tumour immune evasion through glycolytic reprogramming; moreover, Dac51 treatment is synergistic with anti-PD-L1 therapy [108]. Cai et al. recently discovered that the augmentation of HCC cell glycolysis by circular RHBBD1 restrained PD-1-targeted therapy through increased PIK3R1 translation induced by YTHDF1 [109]. That study revealed that targeting circRHBDD1/YTHDF1/PIK3R1 in HCC has an immune-enhancing effect (Fig. 2). These results prove that reprogramming the RNA epitranscriptome is a potential strategy for immunotherapy.

#### Lipid metabolism

Lipid metabolism reshapes the TIME and regulates immune responses, affecting tumour immune escape [150]. Fatty acid (FA) catabolism favours the effector function of CD8^+^ T cells in the TIME [151]. Fatty acid oxidation (FAO) inhibits the activation of tumour-killing T effector cells (Teffs) while promoting the proliferation of Tregs [152]. Cholesterol regulates immune function in multiple ways. A high cholesterol content in the TIME induces T-cell exhaustion and is correlated with the upregulation of immune-inhibitory molecules [153]. The inhibition of cholesterol esterification potentiates the effector function of CD8^+^ T cells [154]. Additionally, the increased levels of citrate and succinate resulting from the tricarboxylic acid (TCA) cycle bolster the effector function of DCs and macrophages [155]. Tumour lipid peroxidation drives ferroptosis, and dysregulated lipid metabolism in ferroptotic cancer cells modulates tumour immunity [156]. Lipid metabolites released from ferroptotic cancer cells drive immune cells to mobilize within tumour cells. The released oxidized lipid mediators hydroxyeicosatetraenoic acids (HETEs) activate anti-tumour immunity by recruiting immune cells to find ferroptotic tumour cells. PEG2 derived from lipid metabolism suppresses the tumoricidal effect of NK cells, cytotoxic T cells, and conventional type 1 DCs, mediating immune evasion [157]. Immune cells also undergo ferroptosis with lipid peroxidation, affecting anti-tumour immunity.

Mounting evidence indicates that m^6^A-driven lipid metabolism reprogramming is associated with multiple metabolic diseases [158, 159]. Recent studies have investigated m^6^A methylation in tumour lipid metabolism. m^6^A methylation regulates lipid accumulation in HepaRG and HepG2 liver cancer cells by affecting carboxylesterase 2 (CES2) expression in a YTHDC2-dependent manner. Depletion of METTL3 and METTL14 increased CES2 expression and attenuated lipid accumulation, while knockout of FTO or ALKBH5 affected CES2 expression and lipid accumulation in a reversible manner [110]. Another study reported that METTL3 enhanced the expression of the lipogenesis-related lncRNA LINC00958 in HCC, and the latter promoted lipogenesis and tumour progression [111]. The m^6^A eraser FTO is a lipid metabolism-associated protein and has been proven to induce lipid droplet formation in esophageal cancer cells [112]. In addition, delta 4-desaturase sphingolipid (DEGS2) dysregulation in CRC mediated by m^6^A methylation induces lipid dysregulation and CRC carcinogenesis [113]. Ferroptosis-associated lipid peroxidation in tumours and immune cells modulates anti-tumour immunity and is a pivotal mechanism of immune evasion that has been investigated in recent years [160–162]. Studies have shown that m^6^A methylation regulates lipid peroxidation and ferroptosis through the ferroptosis defence system X_c_^−^, comprising 2 subunits, SLC7A11 and SLC3A4, and a key enzyme of lipid metabolism, ACSL4, which induces ferroptosis in tumours. In GBM, m^6^A binds to NF-κB activating protein (NKAP) to stimulate SLC7A11 splicing and maturation, inhibiting cancer cell ferroptosis, and m^6^A methylation suppression or METTL3 deletion disrupts the ferroptosis-suppressing effect. NKAP also alters the TIME in GBM and affects lipid peroxidation in T cells [114]. That study implied the potential connection between m^6^A, lipid metabolism, and tumour immunity. m^6^A methylation-repressed tumour ferroptosis also depends on the inhibition of SLC7A11 deadenylation and enhancement of SLC7A11 stabilization [115, 116]. Notably, FTO-induced hypomethylation drives the downregulation of SLC7A11 expression, promoting tumour ferroptosis [117]. METTL14 inhibits SLC7A11 expression through YTHDF2-mediated degradation, which can be abrogated by hypoxia [118]. Additionally, YTHDC2 serves as an endogenous ferroptosis inducer due to its inhibition of SLC3A2 [119]. A systematic analysis identified that YTHDF2 interacts with the hypoxia-induced lncRNA-CBSLR to mediate the instability of CBS mRNA, decreasing ACSL4 methylation and leading to the protection of GC cells from ferroptosis and chemoresistance [120]. These data lay a solid foundation for the identification of indispensable m^6^A regulators in tumour immunity. Nevertheless, more convincing data are needed to identify the direct link between m^6^A-regulated lipid metabolic reprogramming in the TIME and immune evasion.

#### AA metabolism

AAs provide essential substances for cancer cell proliferation and regulation of the immune system. Aberrant metabolism of AAs affects anti-tumour immunity and facilitates tumour immune evasion [163]. Methionine is an essential AA, and dietary methionine deficiency suppresses tumour growth [164]. In the TIME, tumour cells restrict anti-tumour immunity through the disruption of methionine metabolism in T cells. It has been revealed that the augmentation of the tumour methionine transporter SLC43A2 disrupts T-cell acquisition of methionine, alters histone methylation, and decreases STAT5 expression, disrupting the cytotoxicity of T cells [165]. Additionally, increased levels of the methionine metabolites 5-methylthioadenosine (MTA) and S-adenosylmethionine (SAM) promote T-cell exhaustion [166]. These published data highlight a critical role of methionine metabolism in reshaping the TIME and tumour immunity. Similarly, several AAs also regulate the plasticity of the TIME and affect tumour immune evasion in a metabolic manner. Glutamine catabolism promotes M2 macrophage activation with the epigenetic control of M2 genes [167]. Glutamine-addicted tumour cells induce IL-23 secretion by TAMs and subsequently drive Treg expansion and tumour-killing lymphocyte suppression, therefore facilitating tumour escape from immune surveillance [168]. Additionally, glutamine metabolism is closely associated with PD-L1 expression. Glutamine deprivation in tumours augments PD-L1 expression, which returns to normal after glutamine recovery [169]. Recent studies have highlighted the promising efficacy of targeting glutamine metabolism in cancer immunotherapy [170]. By using an antagonist of glutamine metabolism, researchers found that tumour glutamine restriction restrains the generation and enrichment of MDSCs in the TIME [171]. Another convincing study revealed that blockade of tumour glutamine metabolism by a glutamine metabolism inhibitor abolished the immunosuppressive TIME, alleviated hypoxia, acidosis, and nutrient deprivation, and restored anti-tumour immunity [172]. Arginine and tryptophan are indispensable for T-cell function, and their metabolism results in AA depletion and metabolite accumulation to inhibit the immune responses of T cells and facilitate tumour immune evasion [163]. In the TIME, TAMs, MDSCs, tolerogenic DCs, and Tregs restrain the arginine accessibility of T cells by expressing high levels of Arg I and arginine transporters. The nitric oxide generated by Arg consumption impairs T cell proliferation and activation. Tryptophan degradation by IDO1/TDO1 leads to Trp deficiency in T cells and thereby blocks the T-cell cycle. The major metabolite from Trp catabolism, kynurenine (Kyn), activates AHR, driving Treg differentiation and suppressing anti-tumour immunity [173]. Targeting AA metabolism in immunotherapy has been increasingly investigated at the preclinical and clinical stages in recent years, yet most studies have failed to show efficacy [174, 175]. The interplay between tumours and immune cells through metabolic regulators contributes to the heterogeneity of the TIME, and the intricate mechanisms by which metabolic players reshape the TIME and regulate tumour immune evasion need to be further clarified.

m^6^A methylation has been found to be a critical mechanism in AA metabolism [121]. Numerous studies have revealed a role of m^6^A-mediated AA metabolic reprogramming in cancer progression and chemoresistance. Glutaminolysis blockade enhanced FTO expression and disrupted YTHDF2-mediated RNA decay of activating transcription factor 4 (ATF4), stimulating autophagy activation and compromising anti-tumour efficacy [121]. FTO contributes to the loss of the von Hippel-Lindau (VHL) tumour suppressor in clear cell renal cell carcinoma (ccRCC). Instead of acting in a manner dependent on the HIF-induced proangiogenic effect, FTO targets the glutamine transporter SLC1A5, modulating metabolic reprogramming and maintaining the survival of VHL-deficient tumour cells [122]. High expression of YTHDF1 in human colon tumour cells causes cisplatin resistance through the enhancement of glutaminase synthesis, and inhibition of glutaminase synergizes with the tumour-suppressing effect of cisplatin [123]. YTHDF1-driven chemoresistance provides a novel strategy to improve clinical chemotherapy. As mentioned previously, methionine metabolism has a direct link with m^6^A methylation; it produces SAM and provides the methyl group for methylation. Methionine regulates the TIME and mediates tumour evasion [165, 166]. Although increasing amounts of relevant data are being reported, there is limited evidence to show the role of m^6^A-modulated AA metabolism in the TIME and immune evasion. A recently published study revealed a novel role of m^6^A methylation-regulated methionine metabolic reprogramming in anti-tumour immunity and tumour immune evasion. Methionine metabolism promotes the m^6^A methylation of immune checkpoints, including PD-L1 and V-domain Ig suppressor of T cell activation (VISTA), enhancing their expression through YTHDF1. Dietary restriction of methionine or YTHDF1 depletion restores the cytotoxicity of CD8^+^ T cells and enhances the efficacy of PD-1 blockade [124]. This new discovery provides a novel strategy for cancer immunotherapy.

### m^6^A methylation drives an acidic TIME and immune evasion

Lactate acid (LA) is a major metabolite in the TIME, and the augmentation of glycolytic activity in tumour cells induces the accumulation of LA, leading to an acidic TIME. LA serves as a ‘metabolic checkpoint’ that hinders the function of immune cells, triggering a hostile, immunosuppressive TIME and leading to immune evasion [176]. The high content of LA in the TIME impairs the proliferation and activation of T cells and NK cells and impedes the antigen presentation of DCs through the activated LA receptor GPR81 [177, 178]. Additionally, high LA content stimulates M2 macrophage, MDSC, and Treg infiltration [179, 180]. Recently, LA was proven to stimulate Treg expression of PD-1 in the highly glycolytic TIME and control immune responses, resulting in the disruption of PD-1 blockade, and inhibition of LA metabolism in Tregs overcame the resistance of Myc-overexpressing tumour cells to PD-1 blockade [181]. Lactate production is closely linked with m^6^A modification, m^6^A-regulated glycolysis affects the lactate content directly, and modulation of lactate dehydrogenase (LDH) is involved in the process. Recent studies have investigated the role of m^6^A in the lactate-driven TIME and immune evasion. It has been found that ALKBH5 targets the transcript of the lactate acid reporter Mct4 in tumours to elevate mRNA levels, enhancing extracellular lactate concentrations and the recruitment of Tregs and MDSCs in the TIME. ALKBH5 silencing or the use of ALKBH5 inhibitors in tumours improves immunotherapy efficacy [125], implying that ALKBH5 is a promising target for immunotherapy in melanoma, CRC, and perhaps other cancers. Interestingly, in a lactate-enriched TIME, lactylation drove METTL3 upregulation in tumour-infiltrating myeloid (TIM) cells and enhanced the immunosuppressive function of TIM cells through YTHDF1-mediated Jak1 translation and the subsequent phosphorylation of STAT3 [126]. These data reveal the interplay between epigenetic reprogramming and metabolites in the TIME, and further studies are needed to discover novel insights for the development of future immunotherapies.

### m^6^A regulates the immunosuppressive TIME and immune evasion

A highly immunosuppressive TIME is a major feature of almost all tumours [182]. In addition to the m^6^A-contamniated hypoxia and acidity that induce an immunosuppressive TIME, the elevated levels of immune inhibitory molecules and the enrichment of immune suppressor cells in the TIME facilitated by m^6^A methylation hinder tumour-specific immune responses and assist immune evasion [183, 184]. PD-L1 is the primary negative immune regulatory molecule in tumour cells; it is also found in immune cells and has been widely targeted in immunotherapy for its ability to suppress T cells and its contribution to immune evasion [185]. Emerging evidence has revealed that m^6^A regulators modulate PD-L1 expression in numerous tumours, leading to an immunosuppressive TIME and immune escape. METTL3 is upregulated in many types of tumour cells. Consistent with its protumoral role, METTL3 enhances tumour PD-L1 expression based on previous studies. METTL3-mediated m^6^A decoration is essential for promoting tumour PD-L1 expression through IGF2BP1-enhanced RNA stability in bladder cancer, and the activation of JNK signalling augments METTL3 expression [127]. In breast cancer, the METTL3/IGF2BP3 axis inhibits immune surveillance by activating PD-L1 mRNA [128]. Additionally, the mechanisms by which METTL3 regulates PD-L1 expression and thus immune escape have also been investigated in non-small cell lung cancer. METTL3 promotes the circularization of circIGF2BP3 by YTHDC1, and the latter subsequently triggers the deubiquitination of PD-L1, elevating its level in lung cancer cells [129]. These results indicate that m^6^A methylation is a critical driver of PD-L1-induced immunosuppression in the TIME. As another principal component of the m^6^A writer complex that facilitates RNA binding, METTL14 has also been found to regulate m^6^A methylation-driven tumoral PD-L1 expression. The ubiquitination of PD-L1 in cholangiocarcinoma (CCA) is diminished by METTL14 through YTHDF2-mediated inhibition of the RING E3 ubiquitin ligase Siah2, and the deprivation of Siah2 represses T-cell expansion and toxicity by maintaining tumour PD-L1 expression [130]. Peng et al. illustrated that METTL14 facilitates LPS-induced PD-L1 expression in HCC cells, METTL14 upregulation by LPS strengthens the stability of MIR155HG and further regulates the levels of PD-L1 via the miR-223/STAT1 axis [131]. The study revealed the epigenetic mechanism by which LPS promotes PD-L1 expression and immune escape. Since m^6^A modification is reversible, which enables it to precisely regulate tumour processes, the regulatory effect of m^6^A erasers on immune inhibitory molecules has been investigated. In intrahepatic cholangiocarcinoma (ICC), ALKBH5 interacts with tumour PD-L1 to remove m^6^A deposition in the 3’UTR of PD-L1 mRNA, therefore preventing YTHDF2-mediated RNA decay and sustaining PD-L1 expression, which represses the proliferation of cytotoxic T cells [132]. That study also revealed the complicated TIME-regulating role of ALKBH5 by single-cell mass cytometry analysis. ALKBH5 enhances PD-L1 levels in monocytes/macrophages and attenuates the infiltration of MDSCs. Specimen analysis further confirmed that tumours with high levels of ALKBH5 were more sensitive to anti-PD1 therapy. Recently, a novel mechanism by which ALKBH5 drives an immune-inhibitory TIME was revealed by Jin et al. ALKBH5 overexpression in head and neck squamous cell carcinoma hindered RIG-1-mediated IFNα secretion, decreasing tumour-killing immune cell infiltration in the TIME and exerting a tumour-promoting effect [133]. Furthermore, the m^6^A reading protein HNRNPC was identified to mediate the maturation of DDX58, which encodes RIG-1. FTO dysregulation was recently reported to affect the expression of tumour-derived immunosuppressive molecules. FTO overexpression induced by the hypomethylating agent decitabine in AML cells markedly increased the expression of the critical immune checkpoint protein LILRB4, which is 40-50 times more abundant than endogenous PD-L1 and PD-L2 in AML cell lines. FTO inhibition significantly decreased leukaemia stem cell self-renewal by abolishing the increase in LILRB4 expression and sensitized human AML cells to T-cell cytotoxicity. In vivo analysis further confirmed that targeting FTO/m^6^A/LILRB4 overcomes immune evasion in leukaemia [134]. In melanoma, knockdown of FTO promotes YTHDF2-mediated PD-1, CXCR4, and SOX10 mRNA decay, sensitizing melanoma cells to interferon-gamma and anti-PD-1 therapy [135]. The immune-inhibitory role of FTO has also been confirmed in oral cancer. FTO upregulation in arecoline-exposed oral cancer enhanced PD-L1 transcript levels and stability through m^6^A modification and MYC activity [136]. These compelling results reveal that tumours exploit m^6^A methylation and demethylation to promote an immunosuppressive TIME and facilitate escape from immune surveillance. An understanding of the mechanism by which m^6^A regulates PD-L1 expression and promotes an immunosuppressive TIME will provide novel insights to overcome the clinical challenges of immunotherapy.

Enrichment of immune-suppressing cells, including MDSCs, Tregs, and TAMs, and increases in the levels of immune inhibitory cytokines secreted by these immune cells in the TIME exert an inhibitory effect on tumour-killing immune cells, sustaining the immune-hostile TIME and promoting tumorigenesis [186]. m^6^A modification has been closely linked with suppression of immune infiltration in various tumours. Comprehensive analyses of public datasets have led to the characterization of tumour immune infiltration signatures based on m^6^A modification patterns [187], revealing a critical role of m^6^A methylation in reshaping the TIME and regulating immune evasion. Recent studies have convincingly confirmed that m^6^A potentiates theimmunosuppressive TIME and immune escape through the augmentation of immune inhibitory cells. METTL3 in CRC cells enhances the migration of MDSCs and subsequently inhibits CD8^+^ T cells by increasing CXCR1 transcription through m^6^A-induced upregulation of BHLHE41 expression. METTL3-driven immune repression can be reversed by MDSC depletion. METTL3 silencing or pharmacological inhibition improves the efficacy of PD-1 blockade [137]. Notably, m^6^A-regulated IFN responses in GBM reduce Treg infiltration and strengthen the efficacy of ICB by targeting transcription elongation machinery [138], revealing a novel mechanism by which m^6^A regulates the immune-suppressive TIME and the molecular basis of therapy resistance in GBM. The ALKBH5/MAP3K8 axis has been identified to induce PD-L1^+^ macrophage enrichment in HCC, promoting HCC progression and an immunosuppressive TIME [139] (Table 1).

m^6^A methylation is undoubtedly a modulating program that can reshape the TIME under hypoxic stress, as well as by regulating acidity and metabolic reprogramming and altering the immune infiltration of suppressive immune cells and immune checkpoint expression. As a delicate mechanism controlling the above processes in the TIME, m^6^A methylation is beginning to be recognized as a promising target and prognostic marker for improving immunotherapy.

## The m^6^A methylation programme in immune cells

Immune cells are the primary agents that defend and kill tumour cells. However, impaired activation, expansion, and phenotype switching of immune cells can induce an immune-exhausted and suppressive TIME, leading to tumour evasion [188]. Evidence has shown that m^6^A RNA modification in immune cells is indispensable in the regulation of the homeostasis and function of multiple tumour-infiltrating immune cells, reshaping anti-tumour immunity and modulating immune evasion.

### m^6^A methylation is required for T cell homeostasis and functions

T-cell responses are predominant in anti-tumour immunity, and most cancer immunotherapies and strategies to combat immune evasion are focused on reprogramming T cells [189]. Naïve T cells, possessing stem cell-like ability, undergo expansion and differentiate into distinct effector T cells under the effect of cytokines and associated signalling pathways in the microenvironment [190]. With neoantigen stimulation and activation signals, naïve CD4^+^ T cells differentiate into T helper cells to assist and regulate the immune responses of CD8^+^ T cells and B cell activation, while naïve CD8^+^ T cells differentiate into tumour-killing T cells or cytotoxic T cells (CTLs). Apoptosis occurs for most effector T cells, although a few survive and turn into memory T cells. Unstimulated naïve T cells are in a resting state and can be recycled. These processes, including the proliferation and differentiation of naïve T cells, TCR signalling, and T-cell apoptosis, are critical for T-cell homeostasis and tumour-specific adaptive immunity.

**Fig. 3:**
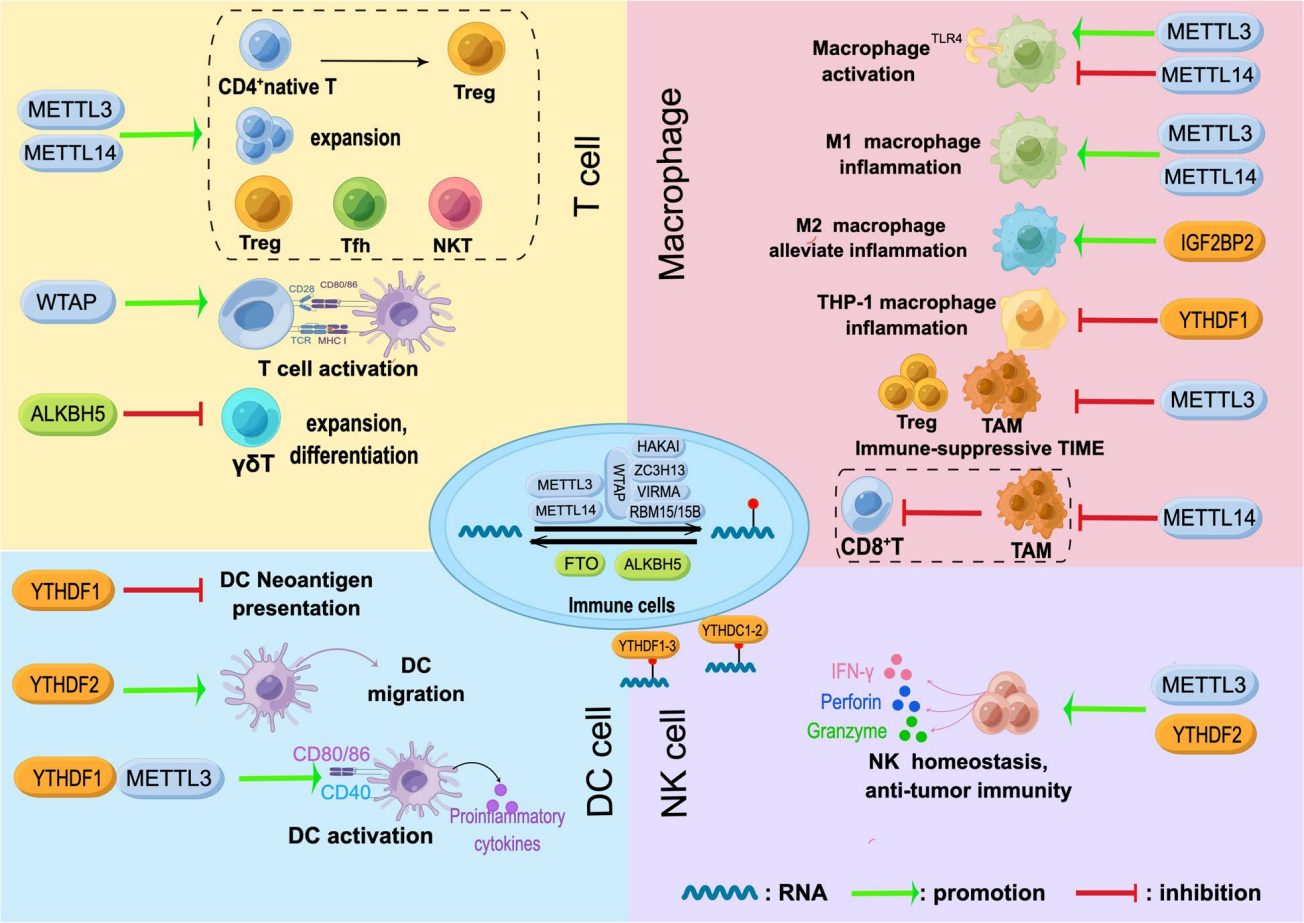
m^6^A modulation in immune cells. m^6^A methylation regulates T-cell homeostasis, macrophage reprogramming (plasticity), and the functions of DCs and NK cells

Increasing evidence has revealed an indispensable role of m^6^A methylation in T-cell homeostasis owing to its regulation of cellular RNA dynamics by affecting the splicing, translation, and degradation of transcripts. The specific mechanism has been investigated in various T cells. Li et al. first reported that m^6^A methylation is critical for the differentiation of naïve CD4^+^ T cells in 2017. Researchers have found that m^6^A methylation is responsible for inducing the RNA decay of SOCS1/3 and CISH in conditional METTL3 and METTL14 knockout mice, safeguarding IL-7/STAT5-mediated T-cell expansion and differentiation [191]. Based on these findings, the team further revealed that METTL3-driven m^6^A methylation is required to maintain Treg suppressive function because it induces an inhibitory effect of SOCS on IL-2/STAT5 signalling [192]. A recent study demonstrated that METTL14 deficiency in T cells was associated with reduced METTL3 abundance, resulting in the failure of naïve T cells transitioning into Tregs and promotion of the Th1/Th17 phenotype [193]. WTAP-driven m^6^A methylation has been proven to modulate TCR signal transduction and control T-cell activation as well as survival [194]. As specialized CD4^+^ effector T cells, T follicular helper (Tfh) cells contribute to antitumour immunity and control PD-L1 blockade efficacy [195, 196]. Evidence has shown that METTL3-induced m^6^A methylation stabilizes the transcripts of predominant Tfh cell signature genes to promote cell differentiation [197]. In addition, m^6^A methylation also participates in VHL-enhanced early-stage Tfh cell differentiation in a glycolytic-epigenetic manner [198]. One recent study focused on the role of m^6^A in the development and function of unconventional T cells. METTL14 deficiency in T cells induced a distinct reduction in invariant NKT (iNKT) cell numbers due to decreased TCR rearrangement. In iNKT cells, loss of METTL14 promoted cell apoptosis, jeopardized cell maturation, and weakened the responses to IL-2/IL-15 TCR stimulation. Impaired cytokine production and TCR signalling are found in mature NKT cells after METTL14 knockdown [199]. γδT cells, another unconventional T lymphocyte, are distributed widely in mucous and provide vital immune surveillance in some tumours due to their non-MHC-restricted antigen recognition [75, 200]. A recent study discovered that deletion of the m^6^A demethylase ALKBH5 in lymphocytes enhances γδT-cell expansion. Mechanistically, increased m^6^A levels in thymocytes reduce the transcript abundance of target genes in Notch signalling, contributing to the augment of γδT cell proliferation and differentiation [201] (Fig. 3). These studies revealed that m^6^A methylation is a critical mechanism of dynamic regulation in T-cell development, differentiation, and antitumour immune responses.

### m^6^A methylation modulates multiple DC immune responses

DCs are the initiators of adaptive immune responses. Unlike other antigen-presenting cells, DCs possess the strongest ability to present antigens that can activate naïve T cells [202], triggering the first anti-tumour immune responses. The dysfunction of DCs leads to immune escape and aggravates carcinogenesis [203]. DCs have been a common topic of immunotherapy in recent years [204]. Recent evidence has shown that m^6^A methylation regulates multiple processes in DC-steered tumour-specific immune responses. m^6^A modification and YTHDF1 regulate the neoantigen presentation of DCs by enhancing the levels of transcripts from lysosome proteases, which increases the expression of neoantigen-disrupting lysosomal cathepsins in DCs. This subsequently results in impaired T-cell activation. The augmentation of antigen cross-presentation and a boost of antigen-specific CD8^+^ T-cell antitumour responses were observed by YTHDF1 depletion in DCs. In addition, YTHDF1-knockout mice exhibited improved PD-1 blockade therapy efficacy [205]. m^6^A modification affects DC migration to draining lymph nodes controlled by CCR7 by altering the metabolic patterns of DCs. By removing m^6^A from the long non-coding RNA lnc-Dpf3 in DCs, CCR7 protects lnc-Dpf3 from YTHDF2-mediated degradation, and this augmentation of lnc-Dpf3 inhibits HIF-1α-driven glycolysis and hinders DC migration [206]. The data provide novel evidence that M^6^A methylation regulates immune homeostasis and metabolic reprogramming. m^6^A methylation also promotes DC activation and maturation. METTL3-mediated methylation and YTHDF1 recognition enhance the expression of the costimulatory molecules CD40, CD80, and CD86 in DCs, as well as the secretion of proinflammatory cytokines, including IL-6, IL-12, and TNF-α [207] (Fig. 3). These findings highlight that m^6^A methylation serves as a critical mechanism regulating the immune responses and homeostasis of DCs.

### m^6^A methylation orchestrates macrophage reprogramming (plasticity)

Macrophages play multiple roles in the host innate immune system and modulate T-cell immunity due to their high plasticity in different microenvironments [208]. Macrophages can be activated through TLR signalling, resulting in increased secretion of cytokines and elevated phagocytosis ability to eliminate infected cells. Macrophages can be switched and repolarized into the M1 and M2 phenotypes, displaying proinflammatory or anti-inflammatory effects. In the TIME, TAMs tend to sustain an immune-suppressive TIME by expressing inhibitory receptors such as PD-L1 and producing IL-10 and TGF-β, inducing T-cell exhaustion and immune escape [28]. With several studies revealing the heterogeneity of macrophages, reprogramming macrophages has been suggested as a novel strategy in immunotherapy. Recent evidence has revealed that m^6^A methylation orchestrates macrophage reprogramming and regulates the TIME. m^6^A methylation modulates dynamic macrophage activation. METTL3-driven methylation positively regulates macrophage activation by accelerating the decay of IRKAM transcripts suppressing TLR signalling [209]. METTL14 maintains the negative feedback control of TLR4/NF-κB signalling by inducing SOCS1 decay, preventing macrophage overactivation [210]. m^6^A methylation promotes macrophage polarization and regulates inflammation. METTL3 facilitates M1 macrophage polarization through m^6^A-mediated augmentation of STAT1 expression [211]. METTL14 deficiency induces M2 macrophage polarization and decreases Myd88 and IL-6 levels due to loss of m^6^A modification [212]. IGF2BP2 targets methylated TSC1 to induce macrophage polarization from the M1 to the M2 phenotype and alleviate inflammation [213]. YTHDF1-induced SOCS3 translation negatively controls inflammatory responses in bacteria-induced THP-1 macrophages [214]. m^6^A methylation regulates TAM reprogramming and immune surveillance. Specific METTL3 knockout in macrophages increases M1- and M2-like TAMs and promotes Treg enrichment into tumours, establishing an immune-suppressive TIME and limiting the efficacy of PD-1 blockade in melanoma. The loss of METTL3 impairs YTHDF1-mediated translation of SPRED2 and leads to the activation of NF-kB and STAT3 signalling through the ERK pathway, promoting M1 and M2 TAM activation and cytokine production [215]. Dong and his colleagues proved that METTL14 deletion in TAMs drives the dysfunction of CD8^+^ T cells. Their investigation revealed that C1q^+^ TAMs were specifically regulated by METTL14, and depletion of METTL14 in C1q^+^ TAMs promoted Ebi3 mRNA accumulation through the METTL14-YTHDF2 axis, resulting in a shift of intratumor CD8^+^ T cells towards a dysfunctional state [216] (Fig. 3). Regulation of m^6^A modification in TAMs is a novel mechanism of immune suppression and tumour immune evasion, suggesting m^6^A-reprogrammed macrophages as a potential target in immunotherapy. m^6^A methylation orchestrates macrophage reprogramming, reinforcing the critical role of epigenetic control in immune surveillance and inflammation-associated diseases.

### m^6^A methylation sustains NK cell homeostasis and its tumoricidal effect

NK cells are a group of cytotoxic cells that play a critical role in immune surveillance [217]. Unlike T-cell activation, NK cell activation can be induced by multiple pathways independent of MHC-restricted antigens [69]. NK cells can directly recognize microbe-infected cells or cancerous cells and mediate a rapid killing effect by releasing perforins, granzyme B, and NK cell cytotoxic factors. Additionally, by producing the cytokines IFN-γ and TGF-β, NK cells can regulate adaptive immunity. Hence, harnessing NK cells is a critical strategy in cancer immunotherapy in addition to T-cell and DC cell-based therapies [218–220]. Recent evidence has revealed a regulatory role of m^6^A methylation in antitumour immunity and the functions of NK cells. METTL3-deficient NK cells exhibit aberrant infiltration in the TIME and disrupt NK cell homeostasis, hindering antitumour immunity [221]. YTHDF2 is essential in NK cell homeostasis, terminal maturation, and antitumour immunity [222]. YTHDF2 reduces the stability of Tardbp RNA, which contributes to IL-15-mediated NK cell proliferation and survival (Fig. 3).

## Conclusions and perspectives

This review describes the m^6^A programme in the TIME and its modulation in immune evasion based on the primary features of the TIME, including hypoxia, metabolic reprogramming, acidity, and immune suppression, and introduces m^6^A modulation in immune cells, highlighting that dynamic and reversible m^6^A methylation is a crucial mechanism reshaping the intricate TIME and regulating immune evasion. Based on extensive evidence, m^6^A methylation is altered under divergent stimuli, which further reshapes the TIME and regulates anti-tumour immunity. These phenomena suggest that m^6^A modulation is highly dependent on biological context, which was demonstrated by He et al. in 2019 [223]. In the hypoxic TIME, the enhancement or restriction of m^6^A regulators by HIF signalling affects tumour-derived RNA fate and subsequently triggers diverse effector functions, supporting a tumour-friendly environment. Notably, hypoxia defines a distinct tumour-regulatory role of ALKBH5 and FTO. Nutrient deprivation and acidity in the TIME also drive m^6^A modulation and affect immune surveillance. Methionine restriction directly results in decreased methylation of immune checkpoint transcripts for insufficient SAM, leading to the restored efficacy of immunotherapy. Lactylation of METTL3 boosts RNA binding ability and m^6^A methylation, enhancing immune-suppressive TIM cells. Although limited evidence has proven that m^6^A regulates lipid metabolism in tumour immunity, numerous clues suggest that future studies should attempt to link m^6^A modification, ferroptosis, and tumour immune surveillance.

m^6^A modulation is a precise modulation in a spatiotemporal manner [224]. m^6^A methylation participates in the specific stage of disease progression [225]; it also displays dynamic alterations in different stages of physiological or pathological processes. As previously discussed, YTHDF2 is essential for the terminal maturation of NK cells rather than functioning in the early stage. Comprehensive analysis revealed that the m^6^A modification pattern was closely related to tumour stage [226]. Hence, further research could consider illustrating the dynamic and reversible m^6^A modulation in different stages of tumour immune surveillance. A specific timepoint or dynamic changes in different stages should be considered to acquire unbiased and accurate results. Novel research tools, such as single-cell RNA sequencing, are preferred for application in m^6^A studies.

With vital roles of m^6^A methylation in tumours and immunoregulation, targeting m^6^A methylation to harness the TIME and overcome immune evasion has been a potential strategy to improve tumour therapy. However, there are also obstacles to the application of targeting m^6^A methylation in tumour therapy. As m^6^A methylation is abundant in diverse physiological and pathological processes, and m^6^A modulation is tissue- and/or cell-specific [227, 228]. Distinct m^6^A modification patterns have been demonstrated in diverse tumours, and there are unique m^6^A modulation patterns in tumour cells and immune cells, which exert distinct effects on immune evasion. These results suggest that specific cells or tissues may need to be targeted in m^6^A-based tumour therapy. Although no relevant clinical data are available yet, in vitro and in vivo attempts showed that developing small molecules that target m^6^A RNA modification proteins is an attractive strategy [125, 229–231]. One study provided solid data on efficacy and toxicity effects [230]. Catalytic inhibition of METTL3 by the small molecule STM2457 specifically inhibited key stem cell populations in AML and prolonged the mouse lifespan without overt toxicity to normal haematopoiesis. A target-based drug design combined with a cell-based screen is popular to develop bioactive small molecules with high selectivity for targets and sensitivity to cells. This step contains the structure optimization of the leading compounds through chemical approaches and pharmacology studies, including vigorous validation of the efficacy and toxicity in vitro and in vivo, selecting the optimum concentration of chemical agents with desired efficacy and lowest toxic effects. Additionally, nanomedicine could be further used to specifically deliver bioactive candidates to tumour cells or immune cells in the TIME [231]. The latest study introduced a nanoplatform that achieved the co-delivery of tumour-associated antigens and FTO inhibitors into tumour-infiltrating dendritic cells (DCs), promoting DC maturation and improving tumour-specific immune responses in vivo and in vitro. Targeting m^6^A methylation in tumour therapy are filled with challenges to overcome but bring hope for future therapy.

In conclusion, m^6^A methylation is an indispensable mechanism in tumour immunoregulation and immune evasion. Targeting m^6^A methylation is a promising strategy to overcome immune escape. We hope this review provides novel insights into m^6^A modulation in immunotherapy and further investigation. However, there is a limitation: our review separately introduced the m^6^A methylation-reshaped TIME under individual stimuli and lacked crosstalk among hypoxia, metabolic reprogramming, acidity, and immunosuppression in the TIME. Further research is expected to provide more in-depth evidence on m^6^A methylation-modulated diseases and therapy.

## Data Availability

Not applicable.

## Abbreviations

m^6^A: N6-methyladenosine
TIME: Tumour immune microenvironment
METTL3: Methyltransferase-like 3
METTL14: Methyltransferase-like 14
WTAP: William tumour 1-associated protein
RBM15: RNA binding motif protein 15
ZC3H13: Zinc-finger CCCH domain-containing protein 13
VIRMA: Vir-like m^6^A methyltransferase associated
TAM: Tumour-associated macrophages
Treg: Regulatory T cell
FTO: Fat-mass and obesity-associated protein
ALKBH5: Alpha-ketoglutarate-dependent dioxygenase ALKB homologue 5
RBPs: RNA-binding proteins
IGF2BP: Insulin-like growth factor 2 mRNA-binding proteins
HIFs: Hypoxia-inducible factors
PD-L1: Programmed death-ligand 1
MDSCs: Myeloid-derived suppressor cells
HCC: Hepatocellular carcinoma
BCSCs: Breast cancer stem cells
ECSCs: Endometrial cancer stem-like cells
CRC: Colorectal cancer
MTA1: Metastasis-associated protein 1
AML: Acute myeloid leukaemia
LUSC: Lung squamous carcinogenesis
SERPINE2: Serpin family E member 2
GBM: Glioblastoma
AA: Amino acid
OXPHOS: Oxidative phosphorylation
DCs: Dendritic cells
NK cells: Natural killer cells
ICBs: Immune checkpoint blockades
RCC: Renal cell carcinoma
GC: Gastric cancer
CK2: Casein kinase 2
BCa: Bladder cancer
FAO: Fatty acid oxidation
Teffs: Tumour-killing T effector cells
TCA: Tricarboxylic acid
HETEs: Hydroxyeicosatetraenoic acids
CES2: Carboxylesterase 2
DEGS2: Delta 4-desaturase sphingolipid
NKAP: NF-κB activating protein
MTA: Methylthioadenosine
SAM: S-adenosylmethionine
Kyn: Kynurenine
ATF4: Activating transcription factor 4
VHL: Von Hippel–Lindau
ccRCC: Clear cell renal cell carcinoma
VISTA: V-domain Ig suppressor of T-cell activation
LA: Lactate acid
LDH: Lactate dehydrogenase
TIM cells: Tumour-infiltrating myeloid cells
CCA: Cholangiocarcinoma
ICC: Intrahepatic cholangiocarcinoma
PUFA: Polyunsaturated fatty acids
CTLs: Cytotoxic T cells
Tfh cells: T follicular helper cells
iNKT cell: Invariant NKT cell

## References

[1] Fu T, Dai LJ, Wu SY, Xiao Y, Ma D, Jiang YZ, Shao ZM (2021). Spatial architecture of the immune microenvironment orchestrates tumor immunity and therapeutic response. J Hematol Oncol.

[2] Zhang Y, Song J, Zhao Z, Yang M, Chen M, Liu C, Ji J, Zhu D (2020). Single-cell transcriptome analysis reveals tumor immune microenvironment heterogenicity and granulocytes enrichment in colorectal cancer liver metastases. Cancer Lett.

[3] Duan Q, Zhang H, Zheng J, Zhang L (2020). Turning cold into hot: firing up the tumor microenvironment. Trends Cancer.

[4] Xiao Y, Ma D, Zhao S, Suo C, Shi J, Xue MZ, Ruan M, Wang H, Zhao J, Li Q (2019). Multi-omics profiling reveals distinct microenvironment characterization and suggests immune escape mechanisms of triple-negative breast cancer. Clin Cancer Res.

[5] Sun YF, Wu L, Liu SP, Jiang MM, Hu B, Zhou KQ, Guo W, Xu Y, Zhong Y, Zhou XR (2021). Dissecting spatial heterogeneity and the immune-evasion mechanism of CTCs by single-cell RNA-seq in hepatocellular carcinoma. Nat Commun.

[6] Pitt JM, Marabelle A, Eggermont A, Soria JC, Kroemer G, Zitvogel L (2016). Targeting the tumor microenvironment: removing obstruction to anticancer immune responses and immunotherapy. Ann Oncol.

[7] Lan Y, Moustafa M, Knoll M, Xu C, Furkel J, Lazorchak A, Yeung TL, Hasheminasab SM, Jenkins MH, Meister S (2021). Simultaneous targeting of TGF-β/PD-L1 synergizes with radiotherapy by reprogramming the tumor microenvironment to overcome immune evasion. Cancer Cell.

[8] Gangoso E, Southgate B, Bradley L, Rus S, Galvez-Cancino F, McGivern N, Güç E, Kapourani CA, Byron A, Ferguson KM (2021). Glioblastomas acquire myeloid-affiliated transcriptional programs via epigenetic immunoediting to elicit immune evasion. Cell.

[9] Topper MJ, Vaz M, Marrone KA, Brahmer JR, Baylin SB (2020). The emerging role of epigenetic therapeutics in immuno-oncology. Nat Rev Clin Oncol.

[10] Gomez S, Tabernacki T, Kobyra J, Roberts P, Chiappinelli KB (2020). Combining epigenetic and immune therapy to overcome cancer resistance. Semin Cancer Biol.

[11] Jung H, Kim HS, Kim JY, Sun JM, Ahn JS, Ahn MJ, Park K, Esteller M, Lee SH, Choi JK (2019). DNA methylation loss promotes immune evasion of tumours with high mutation and copy number load. Nat Commun.

[12] Chen H, Yao J, Bao R, Dong Y, Zhang T, Du Y, Wang G, Ni D, Xun Z, Niu X (2021). Cross-talk of four types of RNA modification writers defines tumor microenvironment and pharmacogenomic landscape in colorectal cancer. Mol Cancer.

[13] Li W, Hao Y, Zhang X, Xu S, Pang D (2022). Targeting RNA N(6)-methyladenosine modification: a precise weapon in overcoming tumor immune escape. Mol Cancer.

[14] Barbieri I, Kouzarides T (2020). Role of RNA modifications in cancer. Nat Rev Cancer.

[15] Moshitch-Moshkovitz S, Dominissini D, Rechavi G (2022). The epitranscriptome toolbox. Cell.

[16] Boulias K, Greer EL. Biological roles of adenine methylation in RNA. Nat Rev Genet. 2022. 10.1038/s41576-022-00534-0. 10.1038/s41576-022-00534-0 PMC9974562 36261710

[17] Dorn LE, Lasman L, Chen J, Xu X, Hund TJ, Medvedovic M, Hanna JH, van Berlo JH, Accornero F (2019). The N(6)-Methyladenosine mRNA methylase METTL3 controls cardiac homeostasis and hypertrophy. Circulation.

[18] Xu W, Li J, He C, Wen J, Ma H, Rong B, Diao J, Wang L, Wang J, Wu F (2021). METTL3 regulates heterochromatin in mouse embryonic stem cells. Nature.

[19] Ramalingam H, Kashyap S, Cobo-Stark P, Flaten A, Chang CM, Hajarnis S, Hein KZ, Lika J, Warner GM, Espindola-Netto JM (2021). A methionine-Mettl3-N(6)-methyladenosine axis promotes polycystic kidney disease. Cell Metab.

[20] Shen S, Zhang R, Jiang Y, Li Y, Lin L, Liu Z, Zhao Y, Shen H, Hu Z, Wei Y, Chen F (2021). Comprehensive analyses of m6A regulators and interactive coding and non-coding RNAs across 32 cancer types. Mol Cancer.

[21] Liu L, Wu Y, Li Q, Liang J, He Q, Zhao L, Chen J, Cheng M, Huang Z, Ren H (2020). METTL3 promotes tumorigenesis and metastasis through BMI1 m(6)A methylation in oral squamous cell carcinoma. Mol Ther.

[22] Wu P, Fang X, Liu Y, Tang Y, Wang W, Li X, Fan Y (2021). N6-methyladenosine modification of circCUX1 confers radioresistance of hypopharyngeal squamous cell carcinoma through caspase1 pathway. Cell Death Dis.

[23] Zhang Y, Kang M, Zhang B, Meng F, Song J, Kaneko H, Shimamoto F, Tang B (2019). m(6)A modification-mediated CBX8 induction regulates stemness and chemosensitivity of colon cancer via upregulation of LGR5. Mol Cancer.

[24] Zhang B, Wu Q, Li B, Wang D, Wang L, Zhou YL (2020). m(6)A regulator-mediated methylation modification patterns and tumor microenvironment infiltration characterization in gastric cancer. Mol Cancer.

[25] Chong W, Shang L, Liu J, Fang Z, Du F, Wu H, Liu Y, Wang Z, Chen Y, Jia S (2021). m(6)A regulator-based methylation modification patterns characterized by distinct tumor microenvironment immune profiles in colon cancer. Theranostics.

[26] Li M, Zha X, Wang S (2021). The role of N6-methyladenosine mRNA in the tumor microenvironment. Biochim Biophys Acta Rev Cancer.

[27] Li X, Ma S, Deng Y, Yi P, Yu J (2022). Targeting the RNA m(6)A modification for cancer immunotherapy. Mol Cancer.

[28] Gu Y, Wu X, Zhang J, Fang Y, Pan Y, Shu Y, Ma P (2021). The evolving landscape of N(6)-methyladenosine modification in the tumor microenvironment. Mol Ther.

[29] Li B, Jiang J, Assaraf YG, Xiao H, Chen ZS, Huang C (2020). Surmounting cancer drug resistance: new insights from the perspective of N(6)-methyladenosine RNA modification. Drug Resist Updat.

[30] Zhao BS, Roundtree IA, He C (2017). Post-transcriptional gene regulation by mRNA modifications. Nat Rev Mol Cell Biol.

[31] Zaccara S, Ries RJ, Jaffrey SR (2019). Reading, writing and erasing mRNA methylation. Nat Rev Mol Cell Biol.

[32] Dixit D, Prager BC, Gimple RC, Poh HX, Wang Y, Wu Q, Qiu Z, Kidwell RL, Kim LJY, Xie Q (2021). The RNA m6A reader YTHDF2 maintains oncogene expression and is a targetable dependency in glioblastoma stem cells. Cancer Discov.

[33] Yang X, Zhang S, He C, Xue P, Zhang L, He Z, Zang L, Feng B, Sun J, Zheng M (2020). METTL14 suppresses proliferation and metastasis of colorectal cancer by down-regulating oncogenic long non-coding RNA XIST. Mol Cancer.

[34] Xiang Y, Laurent B, Hsu CH, Nachtergaele S, Lu Z, Sheng W, Xu C, Chen H, Ouyang J, Wang S (2017). RNA m(6)A methylation regulates the ultraviolet-induced DNA damage response. Nature.

[35] Zhou H, Yin K, Zhang Y, Tian J, Wang S (2021). The RNA m6A writer METTL14 in cancers: roles, structures, and applications. Biochim Biophys Acta Rev Cancer.

[36] He L, Li H, Wu A, Peng Y, Shu G, Yin G (2019). Functions of N6-methyladenosine and its role in cancer. Mol Cancer.

[37] Wei J, He C (2019). Site-specific m(6)A editing. Nat Chem Biol.

[38] Wei J, Liu F, Lu Z, Fei Q, Ai Y, He PC, Shi H, Cui X, Su R, Klungland A (2018). Differential m(6)A, m(6)A(m), and m(1)A demethylation mediated by FTO in the cell nucleus and cytoplasm. Mol Cell.

[39] Su R, Dong L, Li C, Nachtergaele S, Wunderlich M, Qing Y, Deng X, Wang Y, Weng X, Hu C (2018). R-2HG exhibits anti-tumor activity by targeting FTO/m(6)A/MYC/CEBPA signaling. Cell.

[40] Cui Y, Zhang C, Ma S, Li Z, Wang W, Li Y, Ma Y, Fang J, Wang Y, Cao W, Guan F (2021). RNA m6A demethylase FTO-mediated epigenetic up-regulation of LINC00022 promotes tumorigenesis in esophageal squamous cell carcinoma. J Exp Clin Cancer Res.

[41] Tang B, Yang Y, Kang M, Wang Y, Wang Y, Bi Y, He S, Shimamoto F (2020). m(6)A demethylase ALKBH5 inhibits pancreatic cancer tumorigenesis by decreasing WIF-1 RNA methylation and mediating Wnt signaling. Mol Cancer.

[42] Zhang S, Zhao BS, Zhou A, Lin K, Zheng S, Lu Z, Chen Y, Sulman EP, Xie K, Bögler O (2017). m(6)A demethylase ALKBH5 maintains tumorigenicity of glioblastoma stem-like cells by sustaining FOXM1 expression and cell proliferation program. Cancer Cell.

[43] Huang Y, Su R, Sheng Y, Dong L, Dong Z, Xu H, Ni T, Zhang ZS, Zhang T, Li C (2019). Small-molecule targeting of oncogenic FTO demethylase in acute myeloid leukemia. Cancer Cell.

[44] Sun K, Du Y, Hou Y, Zhao M, Li J, Du Y, Zhang L, Chen C, Yang H, Yan F, Su R (2021). Saikosaponin D exhibits anti-leukemic activity by targeting FTO/m(6)A signaling. Theranostics.

[45] Lan Q, Liu PY, Bell JL, Wang JY, Hüttelmaier S, Zhang XD, Zhang L, Liu T (2021). The emerging roles of RNA m(6)A methylation and demethylation as critical regulators of tumorigenesis, drug sensitivity, and resistance. Cancer Res.

[46] Niu Y, Lin Z, Wan A, Chen H, Liang H, Sun L, Wang Y, Li X, Xiong XF, Wei B (2019). RNA N6-methyladenosine demethylase FTO promotes breast tumor progression through inhibiting BNIP3. Mol Cancer.

[47] Ma C, Chang M, Lv H, Zhang ZW, Zhang W, He X, Wu G, Zhao S, Zhang Y, Wang D (2018). RNA m(6)A methylation participates in regulation of postnatal development of the mouse cerebellum. Genome Biol.

[48] Song H, Feng X, Zhang H, Luo Y, Huang J, Lin M, Jin J, Ding X, Wu S, Huang H (2019). METTL3 and ALKBH5 oppositely regulate m(6)A modification of TFEB mRNA, which dictates the fate of hypoxia/reoxygenation-treated cardiomyocytes. Autophagy.

[49] Yang Y, Hsu PJ, Chen YS, Yang YG (2018). Dynamic transcriptomic m(6)A decoration: writers, erasers, readers and functions in RNA metabolism. Cell Res.

[50] Liu T, Wei Q, Jin J, Luo Q, Liu Y, Yang Y, Cheng C, Li L, Pi J, Si Y (2020). The m6A reader YTHDF1 promotes ovarian cancer progression via augmenting EIF3C translation. Nucleic Acids Res.

[51] Du H, Zhao Y, He J, Zhang Y, Xi H, Liu M, Ma J, Wu L (2016). YTHDF2 destabilizes m(6)A-containing RNA through direct recruitment of the CCR4-NOT deadenylase complex. Nat Commun.

[52] Shi H, Wang X, Lu Z, Zhao BS, Ma H, Hsu PJ, Liu C, He C (2017). YTHDF3 facilitates translation and decay of N(6)-methyladenosine-modified RNA. Cell Res.

[53] Li S, Qi Y, Yu J, Hao Y, He B, Zhang M, Dai Z, Jiang T, Li S, Huang F (2022). Nuclear Aurora kinase A switches m(6)A reader YTHDC1 to enhance an oncogenic RNA splicing of tumor suppressor RBM4. Signal Transduct Target Ther.

[54] Zhou B, Liu C, Xu L, Yuan Y, Zhao J, Zhao W, Chen Y, Qiu J, Meng M, Zheng Y (2021). N(6) -Methyladenosine reader protein YT521-B homology domain-containing 2 suppresses liver steatosis by regulation of mRNA stability of lipogenic genes. Hepatology.

[55] Wojtas MN, Pandey RR, Mendel M, Homolka D, Sachidanandam R, Pillai RS (2017). Regulation of m(6)A transcripts by the 3′→5′ RNA helicase YTHDC2 is essential for a successful meiotic program in the mammalian germline. Mol Cell.

[56] Kim GW, Siddiqui A (2021). N6-methyladenosine modification of HCV RNA genome regulates cap-independent IRES-mediated translation via YTHDC2 recognition. Proc Natl Acad Sci U S A.

[57] Tanabe A, Tanikawa K, Tsunetomi M, Takai K, Ikeda H, Konno J, Torigoe T, Maeda H, Kutomi G, Okita K (2016). RNA helicase YTHDC2 promotes cancer metastasis via the enhancement of the efficiency by which HIF-1α mRNA is translated. Cancer Lett.

[58] Wang J, Tan L, Jia B, Yu X, Yao R, OUYang N, Yu X, Cao X, Tong J, Chen T (2021). Downregulation of m(6)A reader YTHDC2 promotes the proliferation and migration of malignant lung cells via CYLD/NF-κB pathway. Int J Biol Sci.

[59] Huang H, Weng H, Sun W, Qin X, Shi H, Wu H, Zhao BS, Mesquita A, Liu C, Yuan CL (2018). Recognition of RNA N(6)-methyladenosine by IGF2BP proteins enhances mRNA stability and translation. Nat Cell Biol.

[60] Choe J, Lin S, Zhang W, Liu Q, Wang L, Ramirez-Moya J, Du P, Kim W, Tang S, Sliz P (2018). mRNA circularization by METTL3-eIF3h enhances translation and promotes oncogenesis. Nature.

[61] Allen BM, Hiam KJ, Burnett CE, Venida A, DeBarge R, Tenvooren I, Marquez DM, Cho NW, Carmi Y, Spitzer MH (2020). Systemic dysfunction and plasticity of the immune macroenvironment in cancer models. Nat Med.

[62] Kearney CJ, Vervoort SJ, Hogg SJ, Ramsbottom KM, Freeman AJ, Lalaoui N, Pijpers L, Michie J, Brown KK, Knight DA (2018). Tumor immune evasion arises through loss of TNF sensitivity. Sci Immunol.

[63] DePeaux K, Delgoffe GM (2021). Metabolic barriers to cancer immunotherapy. Nat Rev Immunol.

[64] Bhandari V, Hoey C, Liu LY, Lalonde E, Ray J, Livingstone J, Lesurf R, Shiah YJ, Vujcic T, Huang X (2019). Molecular landmarks of tumor hypoxia across cancer types. Nat Genet.

[65] Hu G, Ma J, Zhang J, Chen Y, Liu H, Huang Y, Zheng J, Xu Y, Xue W, Zhai W (2021). Hypoxia-induced lncHILAR promotes renal cancer metastasis via ceRNA for the miR-613/206/ 1-1-3p/Jagged-1/Notch/CXCR4 signaling pathway. Mol Ther.

[66] Wei X, Chen Y, Jiang X, Peng M, Liu Y, Mo Y, Ren D, Hua Y, Yu B, Zhou Y (2021). Mechanisms of vasculogenic mimicry in hypoxic tumor microenvironments. Mol Cancer.

[67] Feng X, Zhang H, Meng L, Song H, Zhou Q, Qu C, Zhao P, Li Q, Zou C, Liu X, Zhang Z (2021). Hypoxia-induced acetylation of PAK1 enhances autophagy and promotes brain tumorigenesis via phosphorylating ATG5. Autophagy.

[68] You L, Wu W, Wang X, Fang L, Adam V, Nepovimova E, Wu Q, Kuca K (2021). The role of hypoxia-inducible factor 1 in tumor immune evasion. Med Res Rev.

[69] Ni J, Wang X, Stojanovic A, Zhang Q, Wincher M, Bühler L, Arnold A, Correia MP, Winkler M, Koch PS (2020). Single-cell RNA sequencing of tumor-infiltrating NK cells reveals that inhibition of transcription factor HIF-1α unleashes NK cell activity. Immunity.

[70] Gupta VK, Sharma NS, Durden B, Garrido VT, Kesh K, Edwards D, Wang D, Myer C, Mateo-Victoriano B, Kollala SS (2021). Hypoxia-driven oncometabolite L-2HG maintains stemness-differentiation balance and facilitates immune evasion in pancreatic cancer. Cancer Res.

[71] Miar A, Arnaiz E, Bridges E, Beedie S, Cribbs AP, Downes DJ, Beagrie RA, Rehwinkel J, Harris AL (2020). Hypoxia induces transcriptional and translational downregulation of the type I IFN pathway in multiple cancer cell types. Cancer Res.

[72] Noman MZ, Desantis G, Janji B, Hasmim M, Karray S, Dessen P, Bronte V, Chouaib S (2014). PD-L1 is a novel direct target of HIF-1α, and its blockade under hypoxia enhanced MDSC-mediated T cell activation. J Exp Med.

[73] Chiu DK, Tse AP, Xu IM, Di Cui J, Lai RK, Li LL, Koh HY, Tsang FH, Wei LL, Wong CM (2017). Hypoxia inducible factor HIF-1 promotes myeloid-derived suppressor cells accumulation through ENTPD2/CD39L1 in hepatocellular carcinoma. Nat Commun.

[74] Garcia Garcia CJ, Huang Y, Fuentes NR, Turner MC, Monberg ME, Lin D, Nguyen ND, Fujimoto TN, Zhao J, Lee JJ (2022). Stromal HIF2 regulates immune suppression in the pancreatic cancer microenvironment. Gastroenterology.

[75] Park JH, Kim HJ, Kim CW, Kim HC, Jung Y, Lee HS, Lee Y, Ju YS, Oh JE, Park SH (2021). Tumor hypoxia represses γδ T cell-mediated antitumor immunity against brain tumors. Nat Immunol.

[76] Batie M, Frost J, Frost M, Wilson JW, Schofield P, Rocha S (2019). Hypoxia induces rapid changes to histone methylation and reprograms chromatin. Science.

[77] D'Anna F, Van Dyck L, Xiong J, Zhao H, Berrens RV, Qian J, Bieniasz-Krzywiec P, Chandra V, Schoonjans L, Matthews J (2020). DNA methylation repels binding of hypoxia-inducible transcription factors to maintain tumor immunotolerance. Genome Biol.

[78] Lin Z, Niu Y, Wan A, Chen D, Liang H, Chen X, Sun L, Zhan S, Chen L, Cheng C (2020). RNA m(6) A methylation regulates sorafenib resistance in liver cancer through FOXO3-mediated autophagy. EMBO J.

[79] Zhang C, Samanta D, Lu H, Bullen JW, Zhang H, Chen I, He X, Semenza GL (2016). Hypoxia induces the breast cancer stem cell phenotype by HIF-dependent and ALKBH5-mediated m^6^A-demethylation of NANOG mRNA. Proc Natl Acad Sci U S A.

[80] Chen G, Liu B, Yin S, Li S, Guo Y, Wang M, Wang K, Wan X (2020). Hypoxia induces an endometrial cancer stem-like cell phenotype via HIF-dependent demethylation of SOX2 mRNA. Oncogenesis.

[81] Ruan DY, Li T, Wang YN, Meng Q, Li Y, Yu K, Wang M, Lin JF, Luo LZ, Wang DS (2021). FTO downregulation mediated by hypoxia facilitates colorectal cancer metastasis. Oncogene.

[82] Li Q, Ni Y, Zhang L, Jiang R, Xu J, Yang H, Hu Y, Qiu J, Pu L, Tang J, Wang X (2021). HIF-1α-induced expression of m6A reader YTHDF1 drives hypoxia-induced autophagy and malignancy of hepatocellular carcinoma by promoting ATG2A and ATG14 translation. Signal Transduct Target Ther.

[83] Chen Z, Shao YL, Wang LL, Lin J, Zhang JB, Ding Y, Gao BB, Liu DH, Gao XN (2021). YTHDF2 is a potential target of AML1/ETO-HIF1α loop-mediated cell proliferation in t(8;21) AML. Oncogene.

[84] Xu P, Hu K, Zhang P, Sun ZG, Zhang N (2022). Hypoxia-mediated YTHDF2 overexpression promotes lung squamous cell carcinoma progression by activation of the mTOR/AKT axis. Cancer Cell Int.

[85] Hou J, Zhang H, Liu J, Zhao Z, Wang J, Lu Z, Hu B, Zhou J, Zhao Z, Feng M (2019). YTHDF2 reduction fuels inflammation and vascular abnormalization in hepatocellular carcinoma. Mol Cancer.

[86] Zhu P, He F, Hou Y, Tu G, Li Q, Jin T, Zeng H, Qin Y, Wan X, Qiao Y (2021). A novel hypoxic long noncoding RNA KB-1980E6.3 maintains breast cancer stem cell stemness via interacting with IGF2BP1 to facilitate c-Myc mRNA stability. Oncogene.

[87] Jiang L, Li Y, He Y, Wei D, Yan L, Wen H (2021). Knockdown of m6A reader IGF2BP3 inhibited hypoxia-induced cell migration and angiogenesis by regulating hypoxia inducible factor-1α in stomach cancer. Front Oncol.

[88] Dong F, Qin X, Wang B, Li Q, Hu J, Cheng X, Guo D, Cheng F, Fang C, Tan Y (2021). ALKBH5 facilitates hypoxia-induced paraspeckle assembly and IL8 secretion to generate an immunosuppressive tumor microenvironment. Cancer Res.

[89] Wang Q, Guo X, Li L, Gao Z, Su X, Ji M, Liu J (2020). N(6)-methyladenosine METTL3 promotes cervical cancer tumorigenesis and Warburg effect through YTHDF1/HK2 modification. Cell Death Dis.

[90] Zhang K, Zhang T, Yang Y, Tu W, Huang H, Wang Y, Chen Y, Pan K, Chen Z (2022). N(6)-methyladenosine-mediated LDHA induction potentiates chemoresistance of colorectal cancer cells through metabolic reprogramming. Theranostics.

[91] Shen C, Xuan B, Yan T, Ma Y, Xu P, Tian X, Zhang X, Cao Y, Ma D, Zhu X (2020). m(6)A-dependent glycolysis enhances colorectal cancer progression. Mol Cancer.

[92] Li Z, Peng Y, Li J, Chen Z, Chen F, Tu J, Lin S, Wang H (2020). N(6)-methyladenosine regulates glycolysis of cancer cells through PDK4. Nat Commun.

[93] Ma L, Xue X, Zhang X, Yu K, Xu X, Tian X, Miao Y, Meng F, Liu X, Guo S (2022). The essential roles of m(6)A RNA modification to stimulate ENO1-dependent glycolysis and tumorigenesis in lung adenocarcinoma. J Exp Clin Cancer Res.

[94] Du L, Li Y, Kang M, Feng M, Ren Y, Dai H, Wang Y, Wang Y, Tang B (2021). USP48 is upregulated by Mettl14 to attenuate hepatocellular carcinoma via regulating SIRT6 stabilization. Cancer Res.

[95] Zhang C, Chen L, Liu Y, Huang J, Liu A, Xu Y, Shen Y, He H, Xu D (2021). Downregulated METTL14 accumulates BPTF that reinforces super-enhancers and distal lung metastasis via glycolytic reprogramming in renal cell carcinoma. Theranostics.

[96] Lin JX, Lian NZ, Gao YX, Zheng QL, Yang YH, Ma YB, Xiu ZS, Qiu QZ, Wang HG, Zheng CH (2022). m6A methylation mediates LHPP acetylation as a tumour aerobic glycolysis suppressor to improve the prognosis of gastric cancer. Cell Death Dis.

[97] Ou B, Liu Y, Yang X, Xu X, Yan Y, Zhang J (2021). C5aR1-positive neutrophils promote breast cancer glycolysis through WTAP-dependent m6A methylation of ENO1. Cell Death Dis.

[98] Yu H, Zhao K, Zeng H, Li Z, Chen K, Zhang Z, Li E, Wu Z (2021). N(6)-methyladenosine (m(6)A) methyltransferase WTAP accelerates the Warburg effect of gastric cancer through regulating HK2 stability. Biomed Pharmacother.

[99] Li Y, He L, Wang Y, Tan Y, Zhang F (2022). N(6)-methyladenosine methyltransferase KIAA1429 elevates colorectal cancer aerobic glycolysis via HK2-dependent manner. Bioengineered.

[100] Qing Y, Dong L, Gao L, Li C, Li Y, Han L, Prince E, Tan B, Deng X, Wetzel C (2021). R-2-hydroxyglutarate attenuates aerobic glycolysis in leukemia by targeting the FTO/m(6)A/PFKP/LDHB axis. Mol Cell.

[101] Huang J, Sun W, Wang Z, Lv C, Zhang T, Zhang D, Dong W, Shao L, He L, Ji X (2022). FTO suppresses glycolysis and growth of papillary thyroid cancer via decreasing stability of APOE mRNA in an N6-methyladenosine-dependent manner. J Exp Clin Cancer Res.

[102] Yang X, Shao F, Guo D, Wang W, Wang J, Zhu R, Gao Y, He J, Lu Z (2021). WNT/β-catenin-suppressed FTO expression increases m(6)A of c-Myc mRNA to promote tumor cell glycolysis and tumorigenesis. Cell Death Dis.

[103] Yu H, Yang X, Tang J, Si S, Zhou Z, Lu J, Han J, Yuan B, Wu Q, Lu Q, Yang H (2021). ALKBH5 inhibited cell proliferation and sensitized bladder cancer cells to cisplatin by m6A-CK2α-mediated glycolysis. Mol Ther Nucleic Acids.

[104] Wang Y, Lu JH, Wu QN, Jin Y, Wang DS, Chen YX, Liu J, Luo XJ, Meng Q, Pu HY (2019). LncRNA LINRIS stabilizes IGF2BP2 and promotes the aerobic glycolysis in colorectal cancer. Mol Cancer.

[105] Yao X, Li W, Li L, Li M, Zhao Y, Fang D, Zeng X, Luo Z (2022). YTHDF1 upregulation mediates hypoxia-dependent breast cancer growth and metastasis through regulating PKM2 to affect glycolysis. Cell Death Dis.

[106] Hu Y, Tang J, Xu F, Chen J, Zeng Z, Han S, Wang F, Wang D, Huang M, Zhao Y (2022). A reciprocal feedback between N6-methyladenosine reader YTHDF3 and lncRNA DICER1-AS1 promotes glycolysis of pancreatic cancer through inhibiting maturation of miR-5586-5p. J Exp Clin Cancer Res.

[107] Hou Y, Zhang Q, Pang W, Hou L, Liang Y, Han X, Luo X, Wang P, Zhang X, Li L, Meng X (2021). YTHDC1-mediated augmentation of miR-30d in repressing pancreatic tumorigenesis via attenuation of RUNX1-induced transcriptional activation of Warburg effect. Cell Death Differ.

[108] Liu Y, Liang G, Xu H, Dong W, Dong Z, Qiu Z, Zhang Z, Li F, Huang Y, Li Y (2021). Tumors exploit FTO-mediated regulation of glycolytic metabolism to evade immune surveillance. Cell Metab.

[109] Cai J, Chen Z, Zhang Y, Wang J, Zhang Z, Wu J, Mao J, Zuo X (2022). CircRHBDD1 augments metabolic rewiring and restricts immunotherapy efficacy via m(6)A modification in hepatocellular carcinoma. Mol Ther Oncolytics.

[110] Takemoto S, Nakano M, Fukami T, Nakajima M (2021). m(6)A modification impacts hepatic drug and lipid metabolism properties by regulating carboxylesterase 2. Biochem Pharmacol.

[111] Zuo X, Chen Z, Gao W, Zhang Y, Wang J, Wang J, Cao M, Cai J, Wu J, Wang X (2020). M6A-mediated upregulation of LINC00958 increases lipogenesis and acts as a nanotherapeutic target in hepatocellular carcinoma. J Hematol Oncol.

[112] Duan X, Yang L, Wang L, Liu Q, Zhang K, Liu S, Liu C, Gao Q, Li L, Qin G, Zhang Y (2022). m6A demethylase FTO promotes tumor progression via regulation of lipid metabolism in esophageal cancer. Cell Biosci.

[113] Guo W, Zhang C, Feng P, Li M, Wang X, Xia Y, Chen D, Li J (2021). M6A methylation of DEGS2, a key ceramide-synthesizing enzyme, is involved in colorectal cancer progression through ceramide synthesis. Oncogene.

[114] Sun S, Gao T, Pang B, Su X, Guo C, Zhang R, Pang Q (2022). RNA binding protein NKAP protects glioblastoma cells from ferroptosis by promoting SLC7A11 mRNA splicing in an m(6)A-dependent manner. Cell Death Dis.

[115] Liu L, He J, Sun G, Huang N, Bian Z, Xu C, Zhang Y, Cui Z, Xu W, Sun F (2022). The N6-methyladenosine modification enhances ferroptosis resistance through inhibiting SLC7A11 mRNA deadenylation in hepatoblastoma. Clin Transl Med.

[116] Xu Y, Lv D, Yan C, Su H, Zhang X, Shi Y, Ying K (2022). METTL3 promotes lung adenocarcinoma tumor growth and inhibits ferroptosis by stabilizing SLC7A11 m(6)A modification. Cancer Cell Int.

[117] Ji FH, Fu XH, Li GQ, He Q, Qiu XG (2022). FTO prevents thyroid cancer progression by SLC7A11 m6A methylation in a ferroptosis-dependent manner. Front Endocrinol (Lausanne).

[118] Fan Z, Yang G, Zhang W, Liu Q, Liu G, Liu P, Xu L, Wang J, Yan Z, Han H (2021). Hypoxia blocks ferroptosis of hepatocellular carcinoma via suppression of METTL14 triggered YTHDF2-dependent silencing of SLC7A11. J Cell Mol Med.

[119] Ma L, Zhang X, Yu K, Xu X, Chen T, Shi Y, Wang Y, Qiu S, Guo S, Cui J (2021). Targeting SLC3A2 subunit of system X(C)(−) is essential for m(6)A reader YTHDC2 to be an endogenous ferroptosis inducer in lung adenocarcinoma. Free Radic Biol Med.

[120] Yang H, Hu Y, Weng M, Liu X, Wan P, Hu Y, Ma M, Zhang Y, Xia H, Lv K (2022). Hypoxia inducible lncRNA-CBSLR modulates ferroptosis through m6A-YTHDF2-dependent modulation of CBS in gastric cancer. J Adv Res.

[121] Han S, Zhu L, Zhu Y, Meng Y, Li J, Song P, Yousafzai NA, Feng L, Chen M, Wang Y (2021). Targeting ATF4-dependent pro-survival autophagy to synergize glutaminolysis inhibition. Theranostics.

[122] Xiao Y, Thakkar KN, Zhao H, Broughton J, Li Y, Seoane JA, Diep AN, Metzner TJ, von Eyben R, Dill DL (2020). The m(6)A RNA demethylase FTO is a HIF-independent synthetic lethal partner with the VHL tumor suppressor. Proc Natl Acad Sci U S A.

[123] Chen P, Liu XQ, Lin X, Gao LY, Zhang S, Huang X (2021). Targeting YTHDF1 effectively re-sensitizes cisplatin-resistant colon cancer cells by modulating GLS-mediated glutamine metabolism. Mol Ther Oncolytics.

[124] Li T, Tan YT, Chen YX, Zheng XJ, Wang W, Liao K, et al. Methionine deficiency facilitates antitumour immunity by altering m(6)A methylation of immune checkpoint transcripts. Gut 2023;72:501–11. 10.1136/gutjnl-2022-326928 PMC9933173 35803704

[125] Li N, Kang Y, Wang L, Huff S, Tang R, Hui H, Agrawal K, Gonzalez GM, Wang Y, Patel SP, Rana TM (2020). ALKBH5 regulates anti-PD-1 therapy response by modulating lactate and suppressive immune cell accumulation in tumor microenvironment. Proc Natl Acad Sci U S A.

[126] Xiong J, He J, Zhu J, Pan J, Liao W, Ye H, Wang H, Song Y, Du Y, Cui B (2022). Lactylation-driven METTL3-mediated RNA m(6)A modification promotes immunosuppression of tumor-infiltrating myeloid cells. Mol Cell.

[127] Ni Z, Sun P, Zheng J, Wu M, Yang C, Cheng M, Yin M, Cui C, Wang G, Yuan L (2022). JNK signaling promotes bladder cancer immune escape by regulating METTL3-mediated m6A modification of PD-L1 mRNA. Cancer Res.

[128] Wan W, Ao X, Chen Q, Yu Y, Ao L, Xing W, Guo W, Wu X, Pu C, Hu X (2022). METTL3/IGF2BP3 axis inhibits tumor immune surveillance by upregulating N(6)-methyladenosine modification of PD-L1 mRNA in breast cancer. Mol Cancer.

[129] Liu Z, Wang T, She Y, Wu K, Gu S, Li L, Dong C, Chen C, Zhou Y (2021). N(6)-methyladenosine-modified circIGF2BP3 inhibits CD8(+) T-cell responses to facilitate tumor immune evasion by promoting the deubiquitination of PD-L1 in non-small cell lung cancer. Mol Cancer.

[130] Zheng H, Zheng WJ, Wang ZG, Tao YP, Huang ZP, Yang L, Ouyang L, Duan ZQ, Zhang YN, Chen BN (2022). Decreased expression of programmed death ligand-L1 by seven in absentia homolog 2 in cholangiocarcinoma enhances T-cell-mediated antitumor activity. Front Immunol.

[131] Peng L, Pan B, Zhang X, Wang Z, Qiu J, Wang X, Tang N (2022). Lipopolysaccharide facilitates immune escape of hepatocellular carcinoma cells via m6A modification of lncRNA MIR155HG to upregulate PD-L1 expression. Cell Biol Toxicol.

[132] Qiu X, Yang S, Wang S, Wu J, Zheng B, Wang K, Shen S, Jeong S, Li Z, Zhu Y (2021). M(6)A demethylase ALKBH5 regulates PD-L1 expression and tumor Immunoenvironment in intrahepatic cholangiocarcinoma. Cancer Res.

[133] Jin S, Li M, Chang H, Wang R, Zhang Z, Zhang J, He Y, Ma H (2022). The m6A demethylase ALKBH5 promotes tumor progression by inhibiting RIG-I expression and interferon alpha production through the IKKε/TBK1/IRF3 pathway in head and neck squamous cell carcinoma. Mol Cancer.

[134] Su R, Dong L, Li Y, Gao M, Han L, Wunderlich M, Deng X, Li H, Huang Y, Gao L (2020). Targeting FTO suppresses cancer stem cell maintenance and immune evasion. Cancer Cell.

[135] Yang S, Wei J, Cui YH, Park G, Shah P, Deng Y, Aplin AE, Lu Z, Hwang S, He C, He YY (2019). m(6)A mRNA demethylase FTO regulates melanoma tumorigenicity and response to anti-PD-1 blockade. Nat Commun.

[136] Li X, Chen W, Gao Y, Song J, Gu Y, Zhang J, Cheng X, Ai Y (2022). Fat mass and obesity-associated protein regulates arecoline-exposed oral cancer immune response through programmed cell death-ligand 1. Cancer Sci.

[137] Chen H, Pan Y, Zhou Q, Liang C, Wong CC, Zhou Y, Huang D, Liu W, Zhai J, Gou H (2022). METTL3 inhibits antitumor immunity by targeting m(6)A-BHLHE41-CXCL1/CXCR2 axis to promote colorectal cancer. Gastroenterology.

[138] Qiu Z, Zhao L, Shen JZ, Liang Z, Wu Q, Yang K, Min L, Gimple RC, Yang Q, Bhargava S (2022). Transcription elongation machinery is a druggable dependency and potentiates immunotherapy in glioblastoma stem cells. Cancer Discov.

[139] You Y, Wen D, Zeng L, Lu J, Xiao X, Chen Y, Song H, Liu Z (2022). ALKBH5/MAP3K8 axis regulates PD-L1+ macrophage infiltration and promotes hepatocellular carcinoma progression. Int J Biol Sci.

[140] Long L, Wei J, Lim SA, Raynor JL, Shi H, Connelly JP, Wang H, Guy C, Xie B, Chapman NM (2021). CRISPR screens unveil signal hubs for nutrient licensing of T cell immunity. Nature.

[141] Reinfeld BI, Madden MZ, Wolf MM, Chytil A, Bader JE, Patterson AR, Sugiura A, Cohen AS, Ali A, Do BT (2021). Cell-programmed nutrient partitioning in the tumour microenvironment. Nature.

[142] Guo D, Tong Y, Jiang X, Meng Y, Jiang H, Du L, Wu Q, Li S, Luo S, Li M (2022). Aerobic glycolysis promotes tumor immune evasion by hexokinase2-mediated phosphorylation of IκBα. Cell Metab.

[143] Xia L, Oyang L, Lin J, Tan S, Han Y, Wu N, Yi P, Tang L, Pan Q, Rao S (2021). The cancer metabolic reprogramming and immune response. Mol Cancer.

[144] Xu K, Yin N, Peng M, Stamatiades EG, Shyu A, Li P, Zhang X, Do MH, Wang Z, Capistrano KJ (2021). Glycolysis fuels phosphoinositide 3-kinase signaling to bolster T cell immunity. Science.

[145] Li W, Tanikawa T, Kryczek I, Xia H, Li G, Wu K, Wei S, Zhao L, Vatan L, Wen B (2018). Aerobic glycolysis controls myeloid-derived suppressor cells and tumor immunity via a specific CEBPB isoform in triple-negative breast cancer. Cell Metab.

[146] Gemta LF, Siska PJ, Nelson ME, Gao X, Liu X, Locasale JW, Yagita H, Slingluff CL, Hoehn KL, Rathmell JC, Bullock TNJ (2019). Impaired enolase 1 glycolytic activity restrains effector functions of tumor-infiltrating CD8(+) T cells. Sci Immunol.

[147] Zhang X, Li Y, Ma Y, Yang L, Wang T, Meng X, Zong Z, Sun X, Hua X, Li H (2018). Yes-associated protein (YAP) binds to HIF-1α and sustains HIF-1α protein stability to promote hepatocellular carcinoma cell glycolysis under hypoxic stress. J Exp Clin Cancer Res.

[148] Wang JZ, Zhu W, Han J, Yang X, Zhou R, Lu HC, Yu H, Yuan WB, Li PC, Tao J (2021). The role of the HIF-1α/ALYREF/PKM2 axis in glycolysis and tumorigenesis of bladder cancer. Cancer Commun (Lond).

[149] Xie M, Fu XG, Jiang K (2021). Notch1/TAZ axis promotes aerobic glycolysis and immune escape in lung cancer. Cell Death Dis.

[150] Yu W, Lei Q, Yang L, Qin G, Liu S, Wang D, Ping Y, Zhang Y (2021). Contradictory roles of lipid metabolism in immune response within the tumor microenvironment. J Hematol Oncol.

[151] Zhang Y, Kurupati R, Liu L, Zhou XY, Zhang G, Hudaihed A, Filisio F, Giles-Davis W, Xu X, Karakousis GC (2017). Enhancing CD8(+) T cell fatty acid catabolism within a metabolically challenging tumor microenvironment increases the efficacy of melanoma immunotherapy. Cancer Cell.

[152] Zhang C, Yue C, Herrmann A, Song J, Egelston C, Wang T, Zhang Z, Li W, Lee H, Aftabizadeh M (2020). STAT3 activation-induced fatty acid oxidation in CD8(+) T effector cells is critical for obesity-promoted breast tumor growth. Cell Metab.

[153] Ma X, Bi E, Lu Y, Su P, Huang C, Liu L, Wang Q, Yang M, Kalady MF, Qian J (2019). Cholesterol induces CD8(+) T cell exhaustion in the tumor microenvironment. Cell Metab.

[154] Yang W, Bai Y, Xiong Y, Zhang J, Chen S, Zheng X, Meng X, Li L, Wang J, Xu C (2016). Potentiating the antitumour response of CD8(+) T cells by modulating cholesterol metabolism. Nature.

[155] Fan C, Zhang S, Gong Z, Li X, Xiang B, Deng H, Zhou M, Li G, Li Y, Xiong W (2021). Emerging role of metabolic reprogramming in tumor immune evasion and immunotherapy. Sci China Life Sci.

[156] Li D, Li Y (2020). The interaction between ferroptosis and lipid metabolism in cancer. Signal Transduct Target Ther.

[157] Friedmann Angeli JP, Krysko DV, Conrad M (2019). Ferroptosis at the crossroads of cancer-acquired drug resistance and immune evasion. Nat Rev Cancer.

[158] Yang Y, Cai J, Yang X, Wang K, Sun K, Yang Z, Zhang L, Yang L, Gu C, Huang X (2022). Dysregulated m6A modification promotes lipogenesis and development of non-alcoholic fatty liver disease and hepatocellular carcinoma. Mol Ther.

[159] Peng Z, Gong Y, Wang X, He W, Wu L, Zhang L, Xiong L, Huang Y, Su L, Shi P (2022). METTL3-m(6)A-Rubicon axis inhibits autophagy in nonalcoholic fatty liver disease. Mol Ther.

[160] Liao P, Wang W, Wang W, Kryczek I, Li X, Bian Y, Sell A, Wei S, Grove S, Johnson JK (2022). CD8(+) T cells and fatty acids orchestrate tumor ferroptosis and immunity via ACSL4. Cancer Cell.

[161] Fan F, Liu P, Bao R, Chen J, Zhou M, Mo Z, Ma Y, Liu H, Zhou Y, Cai X (2021). A dual PI3K/HDAC inhibitor induces immunogenic Ferroptosis to potentiate cancer immune checkpoint therapy. Cancer Res.

[162] Stockwell BR, Jiang X (2019). A physiological function for Ferroptosis in tumor suppression by the immune system. Cell Metab.

[163] Wang W, Zou W (2020). Amino acids and their transporters in T cell immunity and cancer therapy. Mol Cell.

[164] Xu Q, Li Y, Gao X, Kang K, Williams JG, Tong L, Liu J, Ji M, Deterding LJ, Tong X (2020). HNF4α regulates sulfur amino acid metabolism and confers sensitivity to methionine restriction in liver cancer. Nat Commun.

[165] Bian Y, Li W, Kremer DM, Sajjakulnukit P, Li S, Crespo J, Nwosu ZC, Zhang L, Czerwonka A, Pawłowska A (2020). Cancer SLC43A2 alters T cell methionine metabolism and histone methylation. Nature.

[166] Hung MH, Lee JS, Ma C, Diggs LP, Heinrich S, Chang CW, Ma L, Forgues M, Budhu A, Chaisaingmongkol J (2021). Tumor methionine metabolism drives T-cell exhaustion in hepatocellular carcinoma. Nat Commun.

[167] Liu PS, Wang H, Li X, Chao T, Teav T, Christen S, Di Conza G, Cheng WC, Chou CH, Vavakova M (2017). α-ketoglutarate orchestrates macrophage activation through metabolic and epigenetic reprogramming. Nat Immunol.

[168] Fu Q, Xu L, Wang Y, Jiang Q, Liu Z, Zhang J, Zhou Q, Zeng H, Tong S, Wang T (2019). Tumor-associated macrophage-derived interleukin-23 interlinks kidney cancer glutamine addiction with immune evasion. Eur Urol.

[169] Byun JK, Park M, Lee S, Yun JW, Lee J, Kim JS, Cho SJ, Jeon HJ, Lee IK, Choi YK, Park KG (2020). Inhibition of glutamine utilization synergizes with immune checkpoint inhibitor to promote antitumor immunity. Mol Cell.

[170] Edwards DN, Ngwa VM, Raybuck AL, Wang S, Hwang Y, Kim LC, Cho SH, Paik Y, Wang Q, Zhang S (2021). Selective glutamine metabolism inhibition in tumor cells improves antitumor T lymphocyte activity in triple-negative breast cancer. J Clin Invest.

[171] Oh MH, Sun IH, Zhao L, Leone RD, Sun IM, Xu W, Collins SL, Tam AJ, Blosser RL, Patel CH (2020). Targeting glutamine metabolism enhances tumor-specific immunity by modulating suppressive myeloid cells. J Clin Invest.

[172] Leone RD, Zhao L, Englert JM, Sun IM, Oh MH, Sun IH, Arwood ML, Bettencourt IA, Patel CH, Wen J (2019). Glutamine blockade induces divergent metabolic programs to overcome tumor immune evasion. Science.

[173] Triplett TA, Garrison KC, Marshall N, Donkor M, Blazeck J, Lamb C, Qerqez A, Dekker JD, Tanno Y, Lu WC (2018). Reversal of indoleamine 2,3-dioxygenase-mediated cancer immune suppression by systemic kynurenine depletion with a therapeutic enzyme. Nat Biotechnol.

[174] Steggerda SM, Bennett MK, Chen J, Emberley E, Huang T, Janes JR, Li W, MacKinnon AL, Makkouk A, Marguier G (2017). Inhibition of arginase by CB-1158 blocks myeloid cell-mediated immune suppression in the tumor microenvironment. J Immunother Cancer.

[175] Muller AJ, Manfredi MG, Zakharia Y, Prendergast GC (2019). Inhibiting IDO pathways to treat cancer: lessons from the ECHO-301 trial and beyond. Semin Immunopathol.

[176] Lundø K, Trauelsen M, Pedersen SF, Schwartz TW (2020). Why Warburg works: lactate controls immune evasion through GPR81. Cell Metab.

[177] Brown TP, Bhattacharjee P, Ramachandran S, Sivaprakasam S, Ristic B, Sikder MOF, Ganapathy V (2020). The lactate receptor GPR81 promotes breast cancer growth via a paracrine mechanism involving antigen-presenting cells in the tumor microenvironment. Oncogene.

[178] Brand A, Singer K, Koehl GE, Kolitzus M, Schoenhammer G, Thiel A, Matos C, Bruss C, Klobuch S, Peter K (2016). LDHA-associated lactic acid production blunts tumor immunosurveillance by T and NK cells. Cell Metab.

[179] Zhang A, Xu Y, Xu H, Ren J, Meng T, Ni Y, Zhu Q, Zhang WB, Pan YB, Jin J (2021). Lactate-induced M2 polarization of tumor-associated macrophages promotes the invasion of pituitary adenoma by secreting CCL17. Theranostics.

[180] Yang X, Lu Y, Hang J, Zhang J, Zhang T, Huo Y, Liu J, Lai S, Luo D, Wang L (2020). Lactate-modulated immunosuppression of myeloid-derived suppressor cells contributes to the radioresistance of pancreatic cancer. Cancer Immunol Res.

[181] Kumagai S, Koyama S, Itahashi K, Tanegashima T, Lin YT, Togashi Y, Kamada T, Irie T, Okumura G, Kono H (2022). Lactic acid promotes PD-1 expression in regulatory T cells in highly glycolytic tumor microenvironments. Cancer Cell.

[182] Zheng Y, Chen Z, Han Y, Han L, Zou X, Zhou B, Hu R, Hao J, Bai S, Xiao H (2020). Immune suppressive landscape in the human esophageal squamous cell carcinoma microenvironment. Nat Commun.

[183] Yi L, Wu G, Guo L, Zou X, Huang P (2020). Comprehensive analysis of the PD-L1 and immune infiltrates of m(6)A RNA methylation regulators in head and neck squamous cell carcinoma. Mol Ther Nucleic Acids.

[184] Zhou Z, Zhang J, Xu C, Yang J, Zhang Y, Liu M, Shi X, Li X, Zhan H, Chen W (2021). An integrated model of N6-methyladenosine regulators to predict tumor aggressiveness and immune evasion in pancreatic cancer. EBioMedicine.

[185] Lv H, Lv G, Chen C, Zong Q, Jiang G, Ye D, Cui X, He Y, Xiang W, Han Q (2021). NAD(+) metabolism maintains inducible PD-L1 expression to drive tumor immune evasion. Cell Metab.

[186] Nirmal AJ, Maliga Z, Vallius T, Quattrochi B, Chen AA, Jacobson CA, Pelletier RJ, Yapp C, Arias-Camison R, Chen YA (2022). The spatial landscape of progression and immunoediting in primary melanoma at single-cell resolution. Cancer Discov.

[187] Zhong J, Liu Z, Cai C, Duan X, Deng T, Zeng G (2021). m(6)A modification patterns and tumor immune landscape in clear cell renal carcinoma. J Immunother Cancer.

[188] Chen YP, Lv JW, Mao YP, Li XM, Li JY, Wang YQ, Xu C, Li YQ, He QM, Yang XJ (2021). Unraveling tumour microenvironment heterogeneity in nasopharyngeal carcinoma identifies biologically distinct immune subtypes predicting prognosis and immunotherapy responses. Mol Cancer.

[189] Si J, Shi X, Sun S, Zou B, Li Y, An D, Lin X, Gao Y, Long F, Pang B (2020). Hematopoietic progenitor kinase1 (HPK1) mediates T cell dysfunction and is a druggable target for T cell-based immunotherapies. Cancer Cell.

[190] Geltink RIK, Kyle RL, Pearce EL (2018). Unraveling the complex interplay between T cell metabolism and function. Annu Rev Immunol.

[191] Li HB, Tong J, Zhu S, Batista PJ, Duffy EE, Zhao J, Bailis W, Cao G, Kroehling L, Chen Y (2017). m(6)A mRNA methylation controls T cell homeostasis by targeting the IL-7/STAT5/SOCS pathways. Nature.

[192] Tong J, Cao G, Zhang T, Sefik E, Amezcua Vesely MC, Broughton JP, Zhu S, Li H, Li B, Chen L (2018). m(6)A mRNA methylation sustains Treg suppressive functions. Cell Res.

[193] Lu TX, Zheng Z, Zhang L, Sun HL, Bissonnette M, Huang H, He C (2020). A new model of spontaneous colitis in mice induced by deletion of an RNA m(6)A methyltransferase component METTL14 in T cells. Cell Mol Gastroenterol Hepatol.

[194] Ito-Kureha T, Leoni C, Borland K, Cantini G, Bataclan M, Metzger RN, Ammann G, Krug AB, Marsico A, Kaiser S (2022). The function of Wtap in N(6)-adenosine methylation of mRNAs controls T cell receptor signaling and survival of T cells. Nat Immunol.

[195] Cui C, Wang J, Fagerberg E, Chen PM, Connolly KA, Damo M, Cheung JF, Mao T, Askari AS, Chen S (2021). Neoantigen-driven B cell and CD4 T follicular helper cell collaboration promotes anti-tumor CD8 T cell responses. Cell.

[196] Niogret J, Berger H, Rebe C, Mary R, Ballot E, Truntzer C, Thibaudin M, Derangère V, Hibos C, Hampe L (2021). Follicular helper-T cells restore CD8(+)-dependent antitumor immunity and anti-PD-L1/PD-1 efficacy. J Immunother Cancer.

[197] Yao Y, Yang Y, Guo W, Xu L, You M, Zhang YC, Sun Z, Cui X, Yu G, Qi Z (2021). METTL3-dependent m(6)A modification programs T follicular helper cell differentiation. Nat Commun.

[198] Zhu Y, Zhao Y, Zou L, Zhang D, Aki D, Liu YC (2019). The E3 ligase VHL promotes follicular helper T cell differentiation via glycolytic-epigenetic control. J Exp Med.

[199] Cao L, Morgun E, Genardi S, Visvabharathy L, Cui Y, Huang H, Wang CR (2022). METTL14-dependent m(6)A modification controls iNKT cell development and function. Cell Rep.

[200] Ribot JC, Lopes N, Silva-Santos B (2021). γδ T cells in tissue physiology and surveillance. Nat Rev Immunol.

[201] Ding C, Xu H, Yu Z, Roulis M, Qu R, Zhou J, Oh J, Crawford J, Gao Y, Jackson R (2022). RNA m(6)A demethylase ALKBH5 regulates the development of γδ T cells. Proc Natl Acad Sci U S A.

[202] Morante-Palacios O, Fondelli F, Ballestar E, Martínez-Cáceres EM (2021). Tolerogenic dendritic cells in autoimmunity and inflammatory diseases. Trends Immunol.

[203] Diamond MS, Lin JH, Vonderheide RH (2021). Site-dependent immune escape due to impaired dendritic cell cross-priming. Cancer Immunol Res.

[204] Harari A, Graciotti M, Bassani-Sternberg M, Kandalaft LE (2020). Antitumour dendritic cell vaccination in a priming and boosting approach. Nat Rev Drug Discov.

[205] Han D, Liu J, Chen C, Dong L, Liu Y, Chang R, Huang X, Liu Y, Wang J, Dougherty U (2019). Anti-tumour immunity controlled through mRNA m(6)A methylation and YTHDF1 in dendritic cells. Nature.

[206] Liu J, Zhang X, Chen K, Cheng Y, Liu S, Xia M, Chen Y, Zhu H, Li Z, Cao X (2019). CCR7 chemokine receptor-inducible lnc-Dpf3 restrains dendritic cell migration by inhibiting HIF-1α-mediated glycolysis. Immunity.

[207] Wang H, Hu X, Huang M, Liu J, Gu Y, Ma L, Zhou Q, Cao X (1898). Mettl3-mediated mRNA m(6)A methylation promotes dendritic cell activation. Nat Commun.

[208] Locati M, Curtale G, Mantovani A (2020). Diversity, mechanisms, and significance of macrophage plasticity. Annu Rev Pathol.

[209] Tong J, Wang X, Liu Y, Ren X, Wang A, Chen Z (2021). Pooled CRISPR screening identifies m(6)A as a positive regulator of macrophage activation. Sci Adv.

[210] Du J, Liao W, Liu W, Deb DK, He L, Hsu PJ, Nguyen T, Zhang L, Bissonnette M, He C, Li YC (2020). N(6)-adenosine methylation of Socs1 mRNA is required to sustain the negative feedback control of macrophage activation. Dev Cell.

[211] Liu Y, Liu Z, Tang H, Shen Y, Gong Z, Xie N, Zhang X, Wang W, Kong W, Zhou Y, Fu Y (2019). The N(6)-methyladenosine (m(6)A)-forming enzyme METTL3 facilitates M1 macrophage polarization through the methylation of STAT1 mRNA. Am J Physiol Cell Physiol.

[212] Zheng Y, Li Y, Ran X, Wang D, Zheng X, Zhang M, Yu B, Sun Y, Wu J (2022). Mettl14 mediates the inflammatory response of macrophages in atherosclerosis through the NF-κB/IL-6 signaling pathway. Cell Mol Life Sci.

[213] Wang X, Ji Y, Feng P, Liu R, Li G, Zheng J, Xue Y, Wei Y, Ji C, Chen D, Li J (2021). The m6A reader IGF2BP2 regulates macrophage phenotypic activation and inflammatory diseases by stabilizing TSC1 and PPARγ. Adv Sci (Weinh).

[214] Li Z, Teng M, Jiang Y, Zhang L, Luo X, Liao Y, Yang B (2022). YTHDF1 negatively regulates Treponema pallidum-induced inflammation in THP-1 macrophages by promoting SOCS3 translation in an m6A-dependent manner. Front Immunol.

[215] Yin H, Zhang X, Yang P, Zhang X, Peng Y, Li D, Yu Y, Wu Y, Wang Y, Zhang J (2021). RNA m6A methylation orchestrates cancer growth and metastasis via macrophage reprogramming. Nat Commun.

[216] Dong L, Chen C, Zhang Y, Guo P, Wang Z, Li J, Liu Y, Liu J, Chang R, Li Y (2021). The loss of RNA N(6)-adenosine methyltransferase Mettl14 in tumor-associated macrophages promotes CD8(+) T cell dysfunction and tumor growth. Cancer Cell.

[217] Wu SY, Fu T, Jiang YZ, Shao ZM (2020). Natural killer cells in cancer biology and therapy. Mol Cancer.

[218] Shimasaki N, Jain A, Campana D (2020). NK cells for cancer immunotherapy. Nat Rev Drug Discov.

[219] Rezvani K, Rouce R, Liu E, Shpall E (2017). Engineering natural killer cells for cancer immunotherapy. Mol Ther.

[220] Xie G, Dong H, Liang Y, Ham JD, Rizwan R, Chen J (2020). CAR-NK cells: a promising cellular immunotherapy for cancer. EBioMedicine.

[221] Song H, Song J, Cheng M, Zheng M, Wang T, Tian S, Flavell RA, Zhu S, Li HB, Ding C (2021). METTL3-mediated m(6)A RNA methylation promotes the anti-tumour immunity of natural killer cells. Nat Commun.

[222] Ma S, Yan J, Barr T, Zhang J, Chen Z, Wang LS, Sun JC, Chen J, Caligiuri MA, Yu J (2021). The RNA m6A reader YTHDF2 controls NK cell antitumor and antiviral immunity. J Exp Med.

[223] Shi H, Wei J, He C (2019). Where, when, and how: context-dependent functions of RNA methylation writers, readers, and erasers. Mol Cell.

[224] Shafik AM, Zhang F, Guo Z, Dai Q, Pajdzik K, Li Y, Kang Y, Yao B, Wu H, He C (2021). N6-methyladenosine dynamics in neurodevelopment and aging, and its potential role in Alzheimer’s disease. Genome Biol.

[225] Si W, Li Y, Ye S, Li Z, Liu Y, Kuang W, Chen D, Zhu M (2020). Methyltransferase 3 mediated miRNA m6A methylation promotes stress granule formation in the early stage of acute ischemic stroke. Front Mol Neurosci.

[226] Li W, Gao Y, Jin X, Wang H, Lan T, Wei M, Yan W, Wang G, Li Z, Zhao Z, Jiang X (2022). Comprehensive analysis of N6-methylandenosine regulators and m6A-related RNAs as prognosis factors in colorectal cancer. Mol Ther Nucleic Acids.

[227] Begik O, Lucas MC, Liu H, Ramirez JM, Mattick JS, Novoa EM (2020). Integrative analyses of the RNA modification machinery reveal tissue- and cancer-specific signatures. Genome Biol.

[228] Zhou C, Molinie B, Daneshvar K, Pondick JV, Wang J, Van Wittenberghe N, Xing Y, Giallourakis CC, Mullen AC (2017). Genome-wide maps of m6A circRNAs identify widespread and cell-type-specific methylation patterns that are distinct from mRNAs. Cell Rep.

[229] Deng LJ, Deng WQ, Fan SR, Chen MF, Qi M, Lyu WY, Qi Q, Tiwari AK, Chen JX, Zhang DM, Chen ZS (2022). m6A modification: recent advances, anticancer targeted drug discovery and beyond. Mol Cancer.

[230] Yankova E, Blackaby W, Albertella M, Rak J, De Braekeleer E, Tsagkogeorga G, Pilka ES, Aspris D, Leggate D, Hendrick AG (2021). Small-molecule inhibition of METTL3 as a strategy against myeloid leukaemia. Nature.

[231] Xiao Z, Li T, Zheng X, Lin L, Wang X, Li B, Huang J, Wang Y, Shuai X, Zhu K (2023). Nanodrug enhances post-ablation immunotherapy of hepatocellular carcinoma via promoting dendritic cell maturation and antigen presentation. Bioact Mater.

